# The Product Science of Electrically Heated Tobacco Products: An Updated Narrative Review of the Scientific Literature

**DOI:** 10.7759/cureus.61223

**Published:** 2024-05-28

**Authors:** Sarah Cordery, Keith Thompson, Matthew Stevenson, Liam Simms, Fiona Chapman, Erika Grandolfo, Layla Malt, Sarah Weaver, Ian M Fearon, Thomas Nahde

**Affiliations:** 1 Group Science and Regulatory Affairs, Imperial Brands Plc., Bristol, GBR; 2 Independent Scientific Consultant, Elucid8 Holdings Ltd., Coleraine, GBR; 3 Scientific Research, whatIF? Consulting Ltd., Harwell, GBR; 4 Group Science and Regulatory Affairs, Imperial Brands Reemtsma, Hamburg, DEU

**Keywords:** tobacco heating products, tobacco harm reduction, smoking, risk reduction, public health, next generation products, heated tobacco products, heat-not-burn tobacco

## Abstract

Heated tobacco products represent a novel category of tobacco products in which a tobacco consumable is heated to a temperature that releases nicotine from the tobacco leaf but not to a temperature sufficient to cause combustion. Heated tobacco products may therefore have the potential to be a less harmful alternative for adult smokers who would otherwise continue to smoke cigarettes, as their use should result in exposure to substantially fewer and lower levels of toxicants. This update represents a two-year extension to our previous narrative review, which covered peer-reviewed journal articles published up to August 31, 2021. The scientific evidence published between 2021 and 2023 continues to indicate that aerosols produced from heated tobacco products contain fewer and substantially lower levels of harmful and potentially harmful constituents and that these observed reductions consistently translate to reduced biological effects in both *in vitro* and *in vivo* toxicological studies. Biomarker and clinical data from studies in which product use is controlled within a clinical setting continue to suggest changes in levels of biomarkers of exposure, biomarkers of potential harm, and clinical endpoints indicating the potential for reduced harm with switching to exclusive use of heated tobacco products in adult smokers. Overall, the available peer-reviewed scientific evidence continues to indicate that heated tobacco products offer promise as a potentially less harmful alternative to cigarettes, and as such, the conclusions of our original narrative review remain valid.

## Introduction and background

The health effects associated with cigarette smoking have been extensively documented by the public health and scientific communities [1-3] as well as documented in large-scale systematic reviews and meta-analyses [4-6]. Smoking is known to be a cause of serious diseases in smokers, including lung cancer, heart disease, and emphysema. Public health experts worldwide have concluded that, while nicotine is addictive and not risk-free, it is the toxicants in cigarette smoke generated by burning tobacco and not the nicotine that is the cause of smoking-related disease. In recent years, product categories have emerged that deliver nicotine without burning tobacco. Tobacco harm reduction (THR) refers to strategies designed to reduce the health risks associated with tobacco smoking, but which may involve continued use of nicotine and/or tobacco [7]. In recent years, next-generation nicotine-delivery products (NGPs) have been developed that deliver nicotine without burning tobacco. Potentially lower-risk NGPs will only achieve their THR potential and demonstrate a health benefit if they meet two basic criteria: the NGPs must be acceptable and satisfying for current adult smokers so that they support their transition away from cigarettes, and the NGPs must have been scientifically demonstrated to be significantly less harmful than cigarettes.

Heated tobacco products (HTPs), also known as ‘heat-not-burn’ products, represent one emerging class of commercially available NGPs. These products heat a portion of refined tobacco in a controlled manner to a temperature that produces an inhalable aerosol that contains nicotine and flavor aromas from the tobacco. Heated tobacco products do not operate at high enough temperatures to burn or combust tobacco. Since heated tobacco products do not burn tobacco, the aerosol contains fewer and substantially lower levels of the harmful chemicals identified in cigarette smoke, including those recognized as being harmful and potentially harmful constituents (HPHCs) [8]. This in turn is expected to result in a reduction in toxicant exposure and a subsequent reduction in toxicity in those smokers who exclusively switch to NGPs from cigarettes or substantially reduce their smoking.

To achieve THR within the population, NGPs must have minimal appeal or use among unintended users. Understanding the appeal of HTPs among those who are nicotine naïve, including both adults and youth, compared with the appeal of these products for adult smokers seeking an alternative to cigarettes, is paramount to assessing their public health potential. It is critically important to appreciate that population-level tobacco harm reduction can only be successfully achieved if a scientifically substantiated reduced-harm product is accepted and used by a large number of adult smokers who would otherwise continue to smoke while ensuring newer smokers, vulnerable populations, and youth do not initiate use.

HTPs developed and/or commercialized by manufacturers have taken three distinct engineering approaches in their design [9,10]:

Aerosol-heated tobacco products (aHTPs): Use of a battery-powered handheld device that heats a liquid consumable to generate a warm aerosol; this aerosol then passes through a tobacco consumable to form a tobacco-containing aerosol. These products may also be known as “hybrid” devices. WITH2 is an example of an aHTP.

Carbon-heated tobacco products (cHTPs): Use of a carbon tip that is lit by the adult smoker with a lighter or match, which heats incoming air, which in turn heats a tobacco-cigarette-like product, forming a tobacco-flavored aerosol containing mainly water, glycerol, nicotine, and volatile tobacco components, as well as quantifiable levels of carbon monoxide. Premier and Eclipse are examples of cHTPs.

Electrically heated tobacco products (eHTPs): Use of a battery-powered handheld device that heats small tobacco consumables (also known as “sticks”) that the adult smoker inserts into the device. The tobacco within the consumable is precisely heated by a number of heating elements or blades within the device to a specific temperature, which is insufficient to support combustion. IQOS, Pulze, and Glo are examples of eHTPs. For the purposes of this review, the abbreviation HTPs should be assumed to exclusively refer to eHTPs unless otherwise indicated.

We have previously published a narrative review detailing the available evidence on HTPs in the scientific literature up to August 2021 relating to the first three components of our bespoke scientific framework: product characterization science, biological science, and clinical science [11]. This review concluded that the totality of the scientific evidence available at that time indicated that HTPs were likely to be significantly less harmful than cigarettes and had a risk profile much closer to that of non-tobacco-containing products, including e-cigarettes. These findings were consistent with the concept of a spectrum of risk for non-combustible tobacco and tobacco-free nicotine products, with no product use and cigarette use representing either end of the continuum and non-combustible tobacco and tobacco-free nicotine products placed in between [12].

Our findings were also supported by the conclusions from a recent UK government review on e-cigarettes and HTPs [13]. This present work represents an update to this original narrative review and includes journal articles published between September 1, 2021, and August 31, 2023, thus providing an additional two years’ worth of scientific data to the evidence base in relation to HTPs. A separate narrative review discussing behavioral science (another component of our scientific framework) in relation to HTPs is currently being conducted. The research question addressed by this narrative review is whether the most up-to-date peer-reviewed scientific evidence on electronic HTPs (eHTPs) supports their potential as a reduced-harm nicotine delivery product compared to cigarettes for adult smokers who would otherwise continue to smoke.

## Review

Methods

Information Sources and Search Terms

An interrogation of the PubMed database [14] was conducted to identify all potentially relevant peer-reviewed journal articles published between September 1st, 2021, and September 1st, 2023, relating to HTPs, using the following six search terms: "heat-not-burn," “heated tobacco," “heated cigarette*," “tobacco heating," “heat tobacco,” and “IQOS” as per the original review [11]. For each search term used, the interrogation was limited to it being present within the title and/or abstract of the journal article. No restriction was placed on the date of publication or the risk of bias associated with the article [15]. Each of these interrogations generated a separate list of journal articles, with these six lists being subsequently combined into a single journal article list to remove multiples of any journal article. This single combined journal article list (n=882) represented the initial starting point for this narrative review.

Study Eligibility Criteria

The title and abstract of these articles were manually screened to identify relevant (n=144) and irrelevant (n=738) articles based on inclusion and exclusion criteria. These are shown in Table 1. Specifically, product characterization studies include those studies quantifying chemical constituents present in HTPs and their aerosols; biological science studies include *in vivo* animal and *in vitro* cell studies; and clinical science studies include those studies in which data is obtained from controlled and 'real-world' use by human users, such as those reported in clinical trials and epidemiology studies.

**Table 1:** Inclusion and exclusion criteria HTPs: heated tobacco products; aHTPs: Aerosol-heated tobacco products; cHTPs: carbon-heated tobacco products

INCLUSION CRITERIA	
1.	Published in English.
2.	Published in peer-reviewed journals.
3.	Articles providing quantitative or qualitative data about product characterization science, biological science, or clinical science.
4.	Published between September 1st, 2021, and August 31st, 2023.
EXCLUSION CRITERIA	
1.	Published exclusively in any language other than English.
2.	Previously identified and included in the original review.
3.	Articles providing quantitative or qualitative data about perception and behavior science or population health science.
4.	Published prior to September 1st, 2021 (unless they were not previously identified for inclusion in the original review).
5.	Articles that included data only in the form of journal abstracts, conference posters, conference proceedings, book chapters, or patents.
6.	Articles that had not completed their peer-review process, provided errata relating to previously published articles or had been retracted.
7.	Articles that only discussed cHTPs.
8.	Articles that only discussed aHTPs.
9.	Articles that provided no discussion or experimental data relating to HTPs.
10.	Editorials or commentaries relating solely to the publication of a separate article.
11.	Articles providing data relating only to the marketing, advertising, or regulation of HTPs or articles providing an analysis of internal industry documents or activities.

Identification of Additional Relevant Articles

Additional relevant peer-reviewed journal articles relating to HTPs, not identified through interrogation of the PubMed database, were subsequently identified through six further processes using the same inclusion and exclusion criteria: manual screening of reference lists of all identified journal articles (n=8); manual screening of study protocols identified in the original review; and cross-linking with Clinicaltrials.gov [16] to determine if results relating to the study protocol have been subsequently published (n=1); manual screening of articles published in Contributions to Tobacco and Nicotine Research [17] from 2021 onwards as this journal is not cited in the PubMed database (n=6); manual screening of the ScienceDirect database [18] (n=3); manual screening of the J-Stage database [19] (n=2); manual screening of Imperial Brands internal databases (n=9).

**Figure 1:**
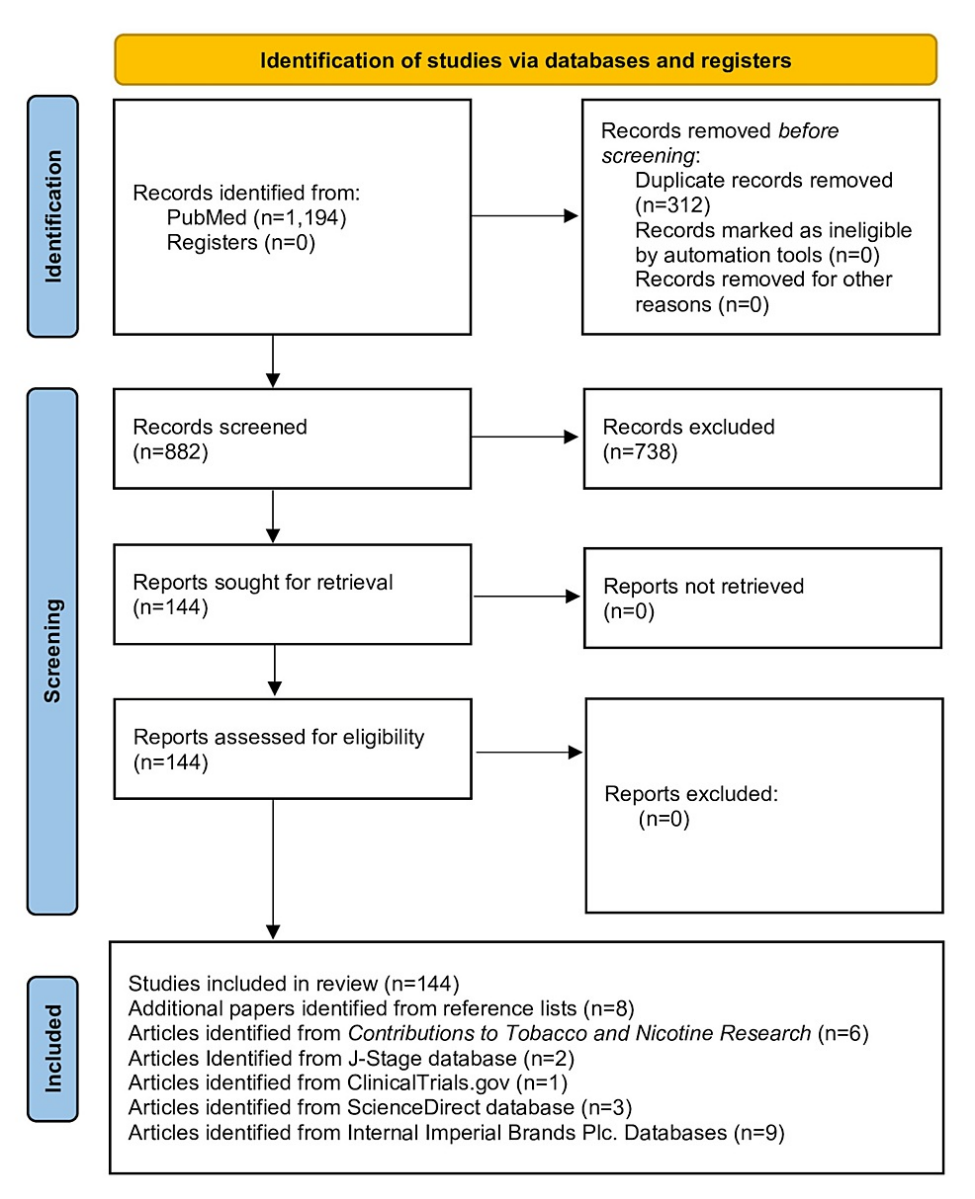
Review methodology

These additional journal articles were then added to those identified from PubMed to create the final list of journal articles referenced in this narrative review (n=173). The overall search strategy and the number of journal articles identified at each stage of the search process are shown in Figure 1.

Aerosol chemistry

This section of the review will discuss those articles that have reported on the chemical characterization of aerosols experimentally produced from HTPs. Areas discussed include harmful and potentially harmful constituents (HPHCs), markers of combustion, toxicological risk exposure and health/cancer risk assessment, radioactivity, and nicotine stereochemistry. With respect to the articles identified that described chemical analyses of HTPs, articles or portions of their results were excluded from detailed discussion in this section of the review if they did not detail a direct quantitative comparison between the HTP(s) under investigation and other nicotine- or tobacco-containing products such as cigarettes and/or EVPs (i.e., quantitative data were reported solely for the HTP(s) investigated) [20-24]; only provided an analysis of chemical constituents present in consumables or only provided an analysis of emissions released by consumables using an experimental methodology which did not involve use of the consumable combined with the operation of the associated handheld device as intended by the manufacturer (i.e. creating an exposure scenario different from that of real-world product use) [25-32]; only provided an analysis of nicotine levels present in HTP aerosol and mainstream cigarette smoke without further detail [33].

Harmful and Potentially Harmful Constituents (HPHCs)

The original review [11] reported that the yields of HPHCs were substantially lower from HTPs than from cigarettes. This observation is supported by the majority of newly identified studies and reviews. Quantified levels of the HPHCs investigated were either substantially lower from HTPs than from cigarettes [34-40] or undetectable or unquantifiable for HTPs [41]. The observed reduction in HPHC yields for HTPs when compared to cigarettes remains even when experimental machine smoking of the products is conducted under a range of extreme conditions of temperature and humidity [36]. A small number of additional identified studies or reviews reported levels of chemical constituents to be higher with HTPs than with cigarettes for some, but not all, of the HTPs investigated [39,42]. Overall, however, as noted by separate authors, “the data demonstrated that IQOS (HTP) generates substantially lower emissions in comparison to cigarette smoke. This indicates that IQOS users are significantly less exposed to gaseous and particulate matter components compared to cigarette users” [37]. The observation of reduced HPHC yields in HTP aerosol when compared to mainstream cigarette smoke was noted to be consistent across studies, irrespective of funding source [43].

Uguna and Snape reported that HPHC yields are often measured under controlled laboratory conditions in which the devices are carefully handled and cleaned following a specific routine [39]. The authors suggest that this scenario may differ from real-world use of the product, where the user may not clean and maintain their device as regularly as suggested by the manufacturer, leading to the potential buildup of material within the device. The authors suggest that continual reheating of this material may result in the generation of higher concentrations of HPHCs and particulate matter than from single-product use [39]. A separate study reported that differences in device cleaning routines (cleaning after the use of a single consumable or after twenty consumables) had no statistically significant effect on emissions of phenol and carbonyl emissions [44]. Based on the available evidence, it is apparent that HPHC levels in HTP aerosols are substantially lower than those present in mainstream cigarette smoke.

Demonstration of the Absence of Combustion-Related Processes in HTPs

The premise of HTPs is that tobacco is heated in a controlled manner, and never burned. Several studies have been conducted to ensure HTPs do not burn tobacco/produce cigarette-like smoke. In agreement with the original review [11], recent studies using a variety of experimental methodologies confirmed the absence of combustion-related solid particles in the aerosol produced from HTPs [37,45-47]. As noted by the authors of one of these studies, “combustion-related solid particles observed in the 3R4F smoke contained elements such as carbon, oxygen, potassium, calcium, and silicon. In contrast, IQOS aerosol particulate matter was composed of semi-volatile organic constituents with some minor traces of oxygen and silicon” [37]. Based on the available data, it is apparent that HTP operations do not involve combustion-related processes.

Identification of (R)-Nicotine in HTP Consumables and Aerosol

Nicotine is an enantiomeric molecule such that it has two stereoisomers: (R)-nicotine and (S)-nicotine. The (S)-variant predominates in tobacco, with only trace levels of (R)-nicotine present. The toxicological significance of (R)-nicotine in tobacco products has not been investigated in detail to date, and therefore it is necessary to ensure that it is present at no more than trace levels and that no significant conversion of (S)-nicotine to (R)-nicotine occurs during the heating process associated with normal HTP use. No information on (R)-nicotine levels in HTPs was identified in the original review [11], so this represents a novel area of research.

A single study was identified that used a liquid chromatography-mass spectrometry methodology to determine the presence of both enantiomeric forms of nicotine in a range of nicotine- and tobacco-containing products, including HTPs [48]. In the study, the reconstituted tobacco present in the consumables and the aerosol produced from them after machine smoking were investigated for two unnamed HTPs. The reconstituted tobacco present in the two consumables contained 0.07% and 0.08% (R)-nicotine, reported as a percentage of total nicotine content. The (R)-nicotine content in HTP aerosol after machine smoking was between 0.59% and 0.91%. These results indicate that while (S)-nicotine remains the overwhelmingly predominant enantiomer present in both the tobacco and the aerosol, there is a small but quantifiable level of enantiomeric interconversion during product use. The levels of (R)-nicotine reported to be present in both HTPs investigated were less than those quantified in the 3R4F Kentucky reference cigarette (0.12% in un-burnt tobacco and between 1.73% and 1.80% in mainstream smoke). The author noted that “this interconversion has the effect of slightly increasing the content of (R)-nicotine in smoke compared with the level in tobacco for combustible cigarettes and HTPs.”

Based on the available evidence, it is apparent that HTP consumables and aerosols contain trace levels of (R)-nicotine and that interconversion to (S)-nicotine may occur with product use. However, the reported levels are substantially lower than those reported for cigarette tobacco and mainstream smoke. The significance and/or toxicological impact of the presence of these trace levels of (R)-nicotine in HTP consumables and aerosols, typically <1% of total nicotine levels, are anticipated to be negligible, but additional research is warranted on this topic.

Presence of Radionuclides in HTPs

The original review did not identify any information in relation to the presence of radionuclides in HTPs. Trace levels of various radionuclides, including polonium-210, a Group 1 carcinogen, have been identified as being present in cigarette tobacco and cigarette smoke [2]. Two studies were identified for inclusion in this update that quantified the levels of radionuclides present in HTPs [49,50].

The authors of the first study determined the levels of polonium-210 (210Po) and lead-210 (210Pb) in the tobacco filler and mainstream smoke of thirteen commercially available brands of cigarettes purchased in Switzerland and aerosol from the IQOS HTP [49]. Polonium-210 levels were roughly comparable when expressed on a mBq per gram of tobacco basis (HTP 23.7±2.1 mBq per gram; cigarettes had a range of 21.0±3.2 to 30.1±2.8 mBq per gram). After machine smoking, 1.8±0.3% of 210Po activity was reported in the aerosol produced from the HTP, while between 9.4±1.4% and 15.6±4.0% of 210Po activity was reported in the mainstream smoke of three of the thirteen cigarettes (quantitative data for the other ten cigarettes was not reported). Lead-210 and polonium-210 were determined to be in temporal equilibrium (210Po/210Pb ratio of 1.06±0.05), indicating comparable levels of lead-210 (210Pb) to polonium-210 (210Po) being present in the products tested. Using the quantitative data produced in their first study, the same researchers estimated that smoking twenty cigarettes per day resulted in an effective annual radiation dose to the lungs of 0.3 mSv per year, while the use of HTP at the same intensity produced a dose of one order of magnitude less (0.03 mSv per year), suggesting a significant decrease in radiation dose to the user with HTP use when compared to cigarette use [50]. As a direct comparison to these values, the worldwide average annual exposure to natural radiation sources has been estimated at 2.4 mSv [51]. Based on these estimated values, it is unlikely that the effective radiation dose due to HTP use is of significant toxicological concern. Based on the available evidence, it is apparent that HTP use may be associated with a lower exposure to radionuclides, as indicated by the annual effective radiation doses, than cigarette smoke. However, further research is warranted to confirm these initial findings.

Toxicological Risk Exposure and Health/Cancer Risk Assessments

Three articles were identified that detailed comparative toxicological exposure assessments using quantitative data relating to the presence of chemical constituents in HTP aerosols and mainstream cigarette smoke [52,53,54]. In these studies, quantitative data for one or more chemical constituents was used to estimate the toxicological risk associated with exposure to the chemical constituent(s) based on several key assumptions, such as the body weight of the user, exposure per day to the chemical constituent(s), and breathing rate of the user. Typically, arbitrary cut-off values were used to indicate whether or not the estimated exposure was regarded as representing a significant increase in harm or not. Different exposure estimates have been reported across these studies based on the endpoints being investigated (cancer- or non-cancer-related endpoints as examples) and the risk assessment approach used. The significance of the reported endpoints and their meanings will be detailed with respect to each article.

The first study used a margin of exposure (MOE) approach for both neurotoxic risk and carcinogenic risk, as well as an incremental lifetime cancer risk (ILCR) approach to estimate the risk associated with exposure to acrylamide present in both HTP aerosol and cigarette smoke [52]. Acrylamide was reported by the authors as being a neurotoxic, genotoxic, and carcinogenic compound that can be generated during heating at high temperatures. The MOE assessment was used to estimate the neurotoxic and carcinogenic risks associated with acrylamide exposure, while the ILCR assessment was used to estimate the lifetime cancer risk. A higher MOE value indicates a greater gap between the estimated exposure level and the level that is believed to cause harm with respect to the endpoint being analyzed. Levels of acrylamide were quantified using gas chromatography-mass spectrometry (GC-MS) and ranged from 235 to 897 ng per cigarette in cigarette smoke. Levels in HTP aerosol ranged between 99 and 187 ng per stick, with median levels in HTP aerosol being approximately one-fifth of those in mainstream smoke. MOE results consistently indicated a low neurotoxic risk for use of either product type, irrespective of gender or daily consumption (which was quantified for between five and 40 cigarettes/HTP consumables per day) and which exceeded the reported minimum safety threshold in all instances. With respect to carcinogenic risk as determined using the MOE approach, HTP aerosol exposure produced higher MOE values than mainstream smoke exposure irrespective of gender and daily consumption for the three ‘benchmark dose lower confidence limit for a 10% response’ (BMDL10) values assessed (0.17, 0.30, and 1.13 mg/kg body weight/day). For the highest value investigated (1.13 mg/kg body weight/day), HTP aerosol exposure exceeded the reported minimum safety threshold, while mainstream smoke did not for all product use intensities investigated. For the ILCR approach, HTP aerosol exposure values were within the acceptable risk zone of between 1x10-4 and 1x10-6 defined by the authors, irrespective of gender and daily consumption, and were consistently lower than the values determined for mainstream smoke. For consumptions of either 20 or 40 consumables per day, the estimated ILCR values exceeded 1x10-5, the cut-off threshold indicated by the authors to represent a negligible cancer risk, whereas lower consumption levels did not. Estimated values for cigarette use exceed this cut-off value for both men and women and for all consumption rates. The authors concluded that “the exposure estimation by tobacco and alternative tobacco products suggested a lower acrylamide-related risk for HTPs than conventional cigarettes, although a potential carcinogenic risk (for HTPs) was observed for moderate consumption.”

The second study estimated hazard index values (HI, a measure of the non-carcinogenic health risk associated with exposure to each constituent) and lifetime cancer risk (LCR) values for twenty-one constituents whose levels were experimentally quantified in mainstream cigarette smoke and HTP aerosol [53]. The HI value for HTP aerosol was 2,449 (based on seven constituents) and 3,609 for mainstream cigarette smoke (based on 20 constituents). A HI value of 1.0 or below was reported as being an acceptable level for no adverse effects on human health. The authors commented that a single constituent, acrolein, had HI values of 2,442 and 3,595 for HTP aerosol and mainstream cigarette smoke, respectively (with this indicating that the other seven constituents in HTP aerosol had a total HI value of approximately 7). The calculated LCR value for cigarettes was the highest, at 2.99x10-4, which was three times higher than that of HTP aerosol (the value was not provided but was approximately 1.0x10-4). A calculated LCR value of 1x10-6 was reported by the authors as indicating an acceptable cancer risk, with a higher LCR value indicating an increase in carcinogenic risk. The authors concluded that “in general, HTPs and e-cigarettes are less harmful than cigarettes.”

The third article reported an estimation of the non-carcinogenic and carcinogenic risks of HTPs and cigarettes calculated using MOE and lifetime excess cancer risk approaches using data obtained from a limited search of the scientific literature [54]. The authors estimated MOE values for twenty-five chemical constituents and lifetime excess cancer risk from ten chemical constituents based on their reported levels in the smoke of different cigarettes and HTP aerosols. The data was provided in qualitative format only. The authors noted that “using available data from HPHC content in conventional cigarette smoke and HTP aerosol, obtained MOE values showed a reduced, but not completely absent risk of HTPs to human health compared to cigarettes for most of the tested compounds” and that “a cancer risk reduction was observed when comparing the mean lifetime excess cancer risk of HTPs with that from cigarette smoke, with a reduction of about one to two orders of magnitude.”

The available evidence from toxicological risk assessments suggests that HTP use may be associated with substantially reduced harm when compared to cigarette use, both when compared to individual chemical constituents and when compared to multiple chemical constituents combined. However, the estimated risk assessment values predict that the harm from HTP use is not zero. It should be noted that toxicological risk assessments have inherent limitations. They assume fixed product use over the course of a lifetime and may overestimate or underestimate their findings in cases of higher or lower than average product consumption, and they do not account for potential poly-product use. Lastly, reporting generalized cancer-related and non-cancer-related risk values does not inform on the risk associated with specific diseases.

Summary of Aerosol Chemistry Data

With respect to chemical constituents, and specifically HPHCs, the newly identified data continues to indicate that HTP aerosols contain fewer and substantially lower levels of these chemical constituents when compared to mainstream cigarette smoke in almost all instances. These conclusions are consistent with those reported in the original review and remain valid.

With respect to toxicological risk assessments, the available evidence indicates that HTP use may be associated with reduced cancer- and non-cancer-related risk when compared to cigarette smoking [52-54]. Separate authors have, however, concluded that the available datasets in relation to HTPs remain limited and conclusions relating to HTPs should be taken with caution [55]. It is apparent that further research is warranted, and additional epidemiological studies into the long-term effects of HTP use may help to determine the absolute cancer risk associated with HTP use [56].

*In Vitro* Toxicology

This section of the review discusses those studies that have investigated the potential *in vitro* toxicological effects of HTPs. For *in vitro* toxicological testing of nicotine- or tobacco-containing products, test material can be generated from whole smoke or aerosol [57]. 

There are three main approaches for testing HTPs *in vitro* [57]:

Direct whole smoke/aerosol analysis: Direct exposure of cells at the air-liquid interface (ALI) or air-agar interface (AAI) to whole smoke or aerosol, which is no more than a few seconds old. The smoke/aerosol contains both particulate and gas-vapor phases and represents the most appropriate experimental surrogate for real-world product use. While this can be regarded as the ‘gold standard’ approach, it is associated with a range of technical and practical challenges, including the absence of standardized procedures [57]. However, several systems have been successfully characterized and/or validated for use with HTPs [58,59]. This method is most suitable for those exposure scenarios where smoke or aerosol can be reasonably expected to come into direct contact with cells under conditions of normal use, such as in the oral cavity and respiratory tract. It is not as relevant for the assessment of systemic endpoints such as cardiovascular or developmental effects, where the other two approaches may be more appropriate as whole smoke or aerosols do not come into direct contact with cells of the cardiovascular system during conditions of normal use.

Bubbled smoke/aerosol trapping: Exposure of cells to a liquid extract produced by bubbling whole smoke or aerosol into phosphate buffered saline (PBS), cell culture medium, or other solvents such as water, which creates a stock solution that can be subsequently serially diluted prior to use in exposing cultured cells.

Fractionated phase sampling: Exposure of cells to a liquid extract produced either from the particulate phase of whole smoke or aerosol collected on a Cambridge filter pad or from bubbling the gas-vapor phase of whole smoke or aerosol into PBS, cell culture medium, or another solvent such as water. These approaches generate fractionated samples for subsequent analysis (i.e., either the particulate phase or the gas-vapor phase are analyzed, but not both, as a single test material). The combination of results obtained from these separate fractionated samples cannot be regarded as an exact representation of the results that would be reasonably expected to be obtained from cells exposed to the first two approaches [57].

When directly comparing these *in vitro* experimental approaches, there is an apparent inverse association between practicability and biological relevance. The necessity of standardized dosimetry approaches to facilitate direct comparisons between studies has also been highlighted [60], and the use of a ‘bridging’ approach to achieve a comparison between new HTP variants and previously characterized ones without the need for repetitive and expensive data generation for each new variant is appropriate [61-63].

With respect to the articles identified that described *in vitro* toxicological analyses of HTPs, articles were excluded from detailed discussion in this section of the review if they did not detail a direct quantitative comparison between the HTP(s) under investigation and other nicotine- or tobacco-containing products such as cigarettes and/or EVPs (i.e., statistical analyses were reported solely between the effects of the HTP and the negative control used) [64,65]; investigated any cell line not related to tissues of the oral cavity, respiratory tract, cardiovascular system or pluripotent stem cells [66-72]; attempted to investigate different human exposure scenarios using the same experimental methodology (i.e., sidestream smoke released by cigarettes being compared to aerosol produced by an HTP as a means of comparing bystander exposure between products) [67].

Cooperation Centre for Scientific Research Relative to Tobacco (CORESTA)-Recommended Assays

The CORESTA, a tobacco testing standards organization, recommends three assays for the *in vitro* toxicological testing of tobacco smoke [73]: Ames Salmonella typhimurium test (bacterial mutagenicity assay); Micronucleus assay and mouse lymphoma assay (mammalian genotoxicity assays); neutral red uptake (NRU) assay (mammalian cytotoxicity assay).

Using these assays, the toxicological effects of particulate phase and gas/vapor phase extracts of the aerosol produced from the DT3.0a HTP (commercialized as Ploom X) were compared to smoke produced from the 1R6F Kentucky reference cigarette [74]. Both regular- and menthol-flavored variants of the DT3.0a HTP were investigated. No mutagenic, genotoxic, or cytotoxic activity was detected for any test samples analyzed from either variant of the DT3.0a HTP, while dose-dependent responses were observed for test samples produced from the 1R6F Kentucky reference cigarette. The authors concluded that “the results of this study indicated that DT3.0a aerosols have chemical and biological properties less likely to be harmful than 1R6F cigarette smoke." Other authors report similar findings. Wang et al. reported that no mutagenic or genotoxic effects were observed for particulate-phase liquid extracts produced from five unnamed HTPs investigated using Ames and micronucleus assays [75]. Chapman et al. exposed cells to HTP aerosol at the ALI and reported significantly reduced or no activity in the NRU assay, micronucleus assay, and Ames test for two prototype HTPs when compared to the 1R6F Kentucky reference cigarette [40].

In the remainder of this section, those studies investigating the potential *in vitro* effects of HTPs using non-CORESTA-recommended assays will be discussed. These studies will be split with respect to the type of cell line used, specifically whether they are derived from either the respiratory tract, oral cavity, or cardiovascular system, followed by further stratification by the test material employed in the study where appropriate.

Cell Lines Modeling the Respiratory Tract

This section will discuss those studies that utilized cells derived from the respiratory tract. A single article was identified that utilized whole smoke/aerosol ALI exposure to compare the *in vitro* effects of HTP aerosol with mainstream cigarette smoke in respiratory tract cells using a 3D human bronchial epithelial model, MucilAir™ [76]. In the study, whole smoke or aerosol was delivered to the 3D model across 28 days (with 16, 32, or 48 diluted puffs per exposure daily session; equivalent to 1.14, 2.29, and 3.43 puffs for 1R6F and 8, 16, and 24 puffs for HTPs). Mainstream cigarette smoke was produced from 1R6F Kentucky reference cigarettes, and HTP aerosol was produced from two prototype devices. Cytotoxicity was quantified by determining lactate dehydrogenase (LDH) release in addition to the quantification of inflammatory cytokine release and cilia active area (that proportion of cilia that are still motile). Compared to HTP aerosol, mainstream cigarette smoke-induced more pronounced effects, which occurred in a dose-dependent manner at earlier time points across all investigated endpoints. The reported differences were observed at a greater dilution for the 1R6F Kentucky reference cigarette smoke (1/14) compared to the HTP aerosols (1/2). The authors concluded that “the findings demonstrate the tobacco harm reduction potential of the prototype heated tobacco products through substantial reductions in toxicological outcomes in *in vitro* 3D human lung models.”

Three studies were identified that utilized bubbled smoke/aerosol extracts to compare the *in vitro* toxicological effects of HTP aerosol with cigarette smoke in respiratory tract cell lines [40,77,78]. In the first study, the effects of liquid extracts of aerosol produced from both the Glo and IQOS HTP on oxidative stress in a human lung fibroblast cell line were investigated [77]. When compared to extracts produced from two cigarettes using the same methodology with a 5% dilution for all extracts, the extracts produced from both HTPs were significantly less toxic than the extracts produced from either cigarette for all endpoints measured. The authors concluded that “heated tobacco product smoke (sic) per se can be toxic despite less toxicity in comparison to cigarettes, which warrants further investigation.”

In the second study, primary human bronchial epithelial cells obtained from a single healthy donor were used in high-content screening (HCS) to investigate the potential toxicological effects of bubbled whole smoke/aerosol extracts produced from two prototype HTPs and the 1R6F Kentucky reference cigarette [40]. HCS was used to assess seven endpoints associated with cellular stress following exposure to bubbled liquid extracts. On an overall level, when results were compared at an equivalent nicotine concentration of 7µg/ml across test materials, the bubbled extract from the 1R6F Kentucky reference cigarette was the most potent test material in the HCS analysis, more than thirteen times more potent than either of the prototype HTP aerosol extracts. The authors concluded that “our findings add to the growing weight of evidence behind the role the HTP category, including the prototype HTPs tested, may play in tobacco harm reduction by providing adult smokers with an acceptable and satisfying reduced harm mode of nicotine delivery compared to combustible cigarettes and give confidence to move into clinical assessment with adult smokers.”

In the third study, human bronchial epithelial (BEAS-2B) cells were used to investigate the effect of dual product use on a range of *in vitro* endpoints, including cell viability and oxidative stress [78]. Liquid extracts were produced by bubbling aerosol or smoke from one IQOS HTP consumable or one Marlboro Red cigarette into phosphate-buffered saline, and the resulting stock solution was used to create serial dilutions for testing. Both were mixed in equivalent volumes to produce a dual extract as a representation of dual product use. Analysis of cell viability indicated that dual extract exposure produced a more pronounced decrease in cell viability than HTP extract alone at concentrations >1%, but a less pronounced decrease than that observed with Marlboro Red extract alone after both 24-hour and 48-hour exposures. Examination of oxidative stress, assessed by quantification of reactive oxygen species (ROS) levels, suggested that both HTP extract and dual extract exposure induced statistically significant increases in ROS levels when compared to controls at concentrations of 0.1%, 1.0%, or 2.5%. It should be noted that, in this study, the Marlboro Red extract did not induce a statistically significant increase in ROS levels compared to controls at any dose levels measured. The HTP extract produced a statistically significant increase in ROS levels at 0.1%, 1.0%, and 5.0%, but not at 2.5%. These findings are unusual and suggestive of issues with the experimental methodology employed. The authors concluded that “the combined exposure of cigarette smoke and IQOS had a negative impact on airway epithelial cell functions *in vitro*.”

Four articles were identified that utilized fractionated sampling liquid extracts to compare the *in vitro* effects of HTPs with cigarettes on respiratory tract cell lines [79-82]. In the first study, the gas-vapor phase of mainstream smoke from three 3R4F Kentucky reference cigarettes and of aerosol from six IQOS HTP consumables was bubbled into PBS to produce extracts [79]. Cytotoxicity was determined in A549 human lung epithelial cells after a 48-hour incubation period with the extracts. The 50% inhibitory concentration (IC50; the concentration required to achieve 50% inhibition of a biological process) with respect to cell viability were 59.7±9.4 puffs/L and 879.1±245.8 puffs/L for the 3R4F and IQOS extracts, respectively, indicating a significantly reduced cytotoxicity of the IQOS extract when compared to the 3R4F extract. Both extracts also produced dose-dependent decreases in total intracellular glutathione levels, quantified as a marker of oxidative stress, after a two-hour incubation period. A one-order-of-magnitude greater concentration of IQOS extract (300 puffs/L) was required to produce a statistically significant difference compared to the control 3R4F extract (30 puffs/L). The authors noted that “significant toxicity was confirmed in heated tobacco product aerosols, albeit to a lesser extent than that in combustible cigarette smoke.”

In the second study, the particulate phase of mainstream smoke from a commercially available cigarette (L&M green label) and of aerosol from the IQOS HTP were dissolved in dimethyl sulfoxide (DMSO) prior to use in cytotoxicity analyses [80]. Cytotoxicity was determined in both human lung adenocarcinoma (Calu-3) and human bronchial epithelial (Beas-2B) cells using MTT and LDH assays. The IC50 values determined in the MTT assay for the cigarette smoke extract were 0.26±0.21 mg/ml and 0.24±0.40 mg/ml in Calu-3 and Beas-2B cells, respectively, while the IC50 values for the HTP extract were above 1 mg/ml in both cell lines. A similar relationship was observed for the LDH assay, although no quantitative data were reported. The authors concluded that “cigarette smoke extracts from traditional cigarettes caused higher cytotoxicity and higher oxidative stress levels than extracts from heated tobacco products in two lung cell lines (Calu-3 and Beas-2B)."

In the third study, the particulate phase of mainstream smoke from the 1R6F Kentucky reference cigarette and of aerosol from three commercially available HTPs (DT3.0a (commercialized as Ploom X), IQOS, and Glo) were dissolved in DMSO prior to use in *in vitro* analyses [81]. Primary human bronchial epithelial cells were used to quantify the effect of exposure to the extracts on activation of the epidermal growth factor receptor (EGFR), with dysregulation of the EGFR signaling pathway being reported to be associated with various chronic diseases, including COPD. The biological sequence from cigarette smoke exposure to EGFR activation involves the induction of oxidative stress by intracellular ROS and the subsequent depletion of glutathione. This causes the EGFR ligand to be secreted and the EGFR to be activated. The 1R6F Kentucky reference cigarette extracts induced all of the tested endpoints, while extracts from the HTPs yielded less pronounced effects at significantly higher concentrations; EGFR phosphorylation (a marker of receptor activation) was not observed even at a five-fold higher concentration for HTPs. The authors concluded that “heated tobacco products are less effective than cigarette smoke to elicit reactive oxygen species-induced EGFR activation.”

In the fourth study, the particulate phase of mainstream smoke from the 1R6F Kentucky reference cigarette and of aerosol from the Ploom S HTP were dissolved in DMSO prior to use in *in vitro* analyses [82]. Human lung adenocarcinoma cells (A549 cells) were used to assess the effect of treatment with particulate matter extracts on DNA methylation and gene transcription. Overall DNA methylation levels were affected by the 1R6F extract but not by the Ploom S extract, while gene expression was affected to a lesser extent by the Ploom S extract than by the 1R6F extract. In a separate study, comparable effects have been observed with respect to DNA methylation in peripheral blood mononuclear cells collected from smokers of cigarettes, HTP users, and non-smokers, where HTP users displayed less pronounced DNA methylation profiles when compared to cigarette smokers [83].

The evidence discussed in this section with respect to cell lines derived from the respiratory tract indicates that test materials derived from HTP aerosols display less pronounced *in vitro* toxicological effects when compared to test materials derived from cigarette smoke across a range of assays. This conclusion is in agreement with that of the original review.

Cell Lines Modeling the Oral Cavity

This section will discuss those studies that utilized cells derived from the oral cavity. Three articles were identified that utilized bubbled liquid extracts to compare the *in vitro* effects of HTPs with cigarettes and/or EVPs in oral cavity cell lines [84-86]. In the first study, bubbled liquid extracts were produced from Marlboro Red cigarettes, an IQOS HTP, and an EVP using a cell culture medium [84]. Two separate oral cell lines were utilized: human gingival fibroblasts and human oral keratinocytes. Cytotoxicity was quantified using the MTT assay, and a scratch assay was also used, which mimics in vivo cell migration during wound healing. All endpoints were evaluated after a 24-hour exposure period for cells to the produced extracts. Undiluted and 50% dilution extracts produced from the cigarette were significantly cytotoxic in gingival fibroblasts, with 67% and 33% inhibition, respectively. In oral keratinocytes, the undiluted extract was significantly cytotoxic (26% inhibition), while other dilutions increased cell viability. Only a single EVP extract (12.5% dilution) produced a statistically significant effect in gingival fibroblasts, with a 26% increase in cell viability. All HTP extract dilutions, with a single exception, increased cell viability in both cell lines. With respect to the scratch wound assay, extracts from both the HTP and EVP did not inhibit wound closure in gingival fibroblasts, while they produced greater cell migration than extracts produced from the cigarette in oral keratinocytes, suggesting more significant progress towards wound closure. The authors concluded that “comparing the different cigarette extracts, tobacco smoke turns out to be the most harmful, e-cigarettes did not determine morphological and functional alterations, and heated tobacco products must be carefully investigated for their possible clinical effects on oral cell populations.”

In the second study, bubbled liquid extracts were produced from an unnamed cigarette and an unnamed HTP using PBS [85]. Primary human oral keratinocytes were utilized, with cell proliferation assessed after six, 12, and 24 hours of exposure to two dilutions of each produced extract (5% and 20%). Cell survival decreased in a dose-dependent manner after exposure to both extract types, compared to controls. There were no statistically significant differences in cell survival between equivalent dilutions at any of the time points investigated. The authors concluded that “heated tobacco products and cigarettes had similar cytotoxic effects." It should be noted that no information was provided in relation to the puff topography profile used or the number of puffs taken on the cigarettes (fourteen puffs were taken on the HTP). A subsequent study by the same research group reported that cell migration in a scratch wound assay (which used a titanium disk as part of an experimental assessment of dental implant wound healing) was significantly inhibited by treatment with both cigarette and HTP bubbled liquid extracts 48 hours after injury with no significant difference between the two product types [86].

The evidence discussed in this section with respect to cell lines derived from the oral cavity does not indicate that test materials derived from HTPs display less pronounced *in vitro* toxicological effects when compared to test materials derived from cigarettes across a range of assays. However, it should be noted that one of the methodologies detailed contains significant experimental limitations [85].

Cell Lines Modeling the Cardiovascular System

This section will discuss those studies that utilized cells derived from the cardiovascular system. Four articles were identified that utilized bubbled liquid extracts to compare the *in vitro* effects of HTPs with cigarettes in cell lines derived from the cardiovascular system [40,87-90]. As mentioned previously, when investigating cardiovascular endpoints, bubbled liquid extract exposure is more reflective of exposure to those constituents that are absorbed into the blood after product use and then maybe transported throughout the cardiovascular system.

In the first study, the effects of liquid extracts produced from three HTPs (IQOS, Glo, and Ploom S) on cytotoxicity in human vascular endothelial cells were compared with those produced by extracts produced from a commercially available cigarette (“hi-lite”) [87]. Cytotoxicity was determined using an MTT assay. For the cigarette, a dose-dependent increase in cytotoxicity was observed for all extract dilutions tested (5% to 80%). Reduced levels of cytotoxicity were observed for the three HTPs, with statistically significant decreases in cytotoxicity being observed for IQOS (10% dilutions and higher) and Ploom S and Glo (40% and 80% dilutions only) when compared to the cigarette. The authors concluded that “the cytotoxicity of heated cigarette smoke (HTP aerosol) extracts was lower than that of burned cigarette smoke extracts in vascular endothelial cells.”

In the second study, the effects of liquid extracts produced from the IQOS 3 DUO and Glo Pro HTPs and the Vype ePen3 EVP in the scratch wound assay were compared [88]. Exposure to 1R6F Kentucky reference cigarette extracts inhibited endothelial cell migration in a dose-dependent fashion, with only the 5% extract dilution permitting complete wound closure after 48 hours. For IQOS, 40% to 80% extract dilutions produced no statistically significant differences in endothelial cell migration when compared to the negative control (cell culture media only), with statistically significant differences observed for the 90% (p=0.009) and 100% (p=0.002) extracts. No statistically significant differences in endothelial cell migration were observed for any extracts produced from the Glo HTP and Vype EVP when compared to negative control. The authors concluded that “we observed a substantial reduction of the effects of aerosol from (the) electronic cigarette and heated tobacco products on endothelial cell migration compared with cigarette smoke.” Additional studies reported comparable effects in relation to wound healing after experimentally induced damage [40,91].

In the third study, the effects of liquid extracts produced from the 1R6F Kentucky reference cigarette, three HTPs (IQOS and two prototype devices), and an EVP were investigated in the Cardio quickPredict assay, which uses human-induced pluripotent stem-cell-derived cardiomyocytes [89]. Liquid extracts produced from the 1R6F Kentucky reference cigarette were deemed to have cardiotoxic potential at a concentration of 0.3 to 0.6% (equivalent to 0.4 to 0.9µg/ml nicotine), while the HTPs demonstrated cardiotoxic potential at a ten-times higher dose (3.3%; 4.1µg/ml nicotine). The EVP extract did not demonstrate any cardiotoxic potential up to the maximum concentration investigated (10%). The authors concluded that “the application of this rapid screening assay to NGP research and the associated findings adds to the weight of evidence indicating that NGPs have a tobacco harm reduction potential when compared to combustible cigarettes." A previous study, using the DevTox quickPredict assay, which screens for early-stage developmental toxicity, determined that liquid extracts from HTPs were significantly less developmentally toxic, with a five-times higher concentration being required to meet the threshold for developmental toxicity in the assay when compared to two Kentucky reference cigarettes, 1R6F and 3R4F [92].

In the fourth study, the effects of liquid extracts produced from the IQOS HTP on a human monocytic (THP-1) cell line were investigated [90]. Whole smoke/aerosol from the 3R4F Kentucky reference cigarette, IQOS HTP, and an EVP (Vype) were bubbled into the cell culture medium. Monocytes were exposed to increasing concentrations of the liquid extracts over 24 hours, with an assessment of cell viability as well as gene and protein expression related to inflammation and oxidative stress. None of the HTP or EVP doses tested decreased cell viability when compared to controls, while all tested doses above 30% of the 3R4F extracts reduced cell viability in a dose-dependent fashion when compared to controls. Both gene and expression levels for the HTP and EVP were significantly reduced when compared to expression levels observed with the 3R4F extracts. The authors concluded that “NGPs overall showed lower responses relative to controls than THP-1 cells exposed to 3R4F aqueous extracts.”

A single study was identified that utilized fractionated sampling liquid extracts to compare the *in vitro* effects of HTPs with cigarettes in a cardiovascular cell line [93]. In this study, the effects of particulate phase extracts produced from three HTPs (Ploom X, IQOS, and Glo) were investigated for their effects on monocyte adhesion to endothelial cells (a key step in the pathogenesis of atherosclerosis) in comparison to the 1R6F Kentucky reference cigarette [93]. The study reported the use of an organ-on-a-chip *in vitro* system, which was reported to mimic major aspects of human physiology. Monocyte adhesion was induced to a significantly lesser extent by extracts produced from the three HTPs than by extracts produced from the 1R6F Kentucky reference cigarette, with comparable results observed between the HTPs. The authors concluded that “our vasculature-on-a-chip model assessed the difference in biological effects between cigarettes and heated tobacco products and suggested a reduced risk potential of heated tobacco products for atherosclerosis.”

The evidence discussed in this section with respect to cell lines derived from the cardiovascular system indicates that test materials derived from HTPs display less pronounced *in vitro* toxicological effects when compared to test materials derived from cigarettes across a range of assays. This conclusion is in agreement with that of the original review.

Assays Modeling a Range of Tissue Types

This section will discuss those studies that utilized multiple cell lines derived from the respiratory tract, cardiovascular system, and oral cavity in the same experimental study design. In the first study, the effects of liquid extracts produced from the 3R4F Kentucky reference cigarette and the IQOS HTP were investigated in the BioMAP Diversity Plus Panel, a commercial product consisting of twelve human primary cell systems containing cell types from multiple tissues with a total of 148 endpoints [94]. The 3R4F extract, at the highest concentration tested of 1%, produced an identifiable toxicity signature, with 22 of the 148 endpoints showing significant activity, while the HTP extract, at the same dose, produced alterations in three of the 148 endpoints. The authors concluded that “data from testing NGP samples did not demonstrate any toxicity signatures at 1% under the conditions of the test."

In the second study, liquid extracts produced from whole smoke and aerosol, as well as gas/vapor phase extracts produced from the 1R6F Kentucky reference cigarette and the Glo HTP, were investigated in the ToxTracker assay, a commercial product that utilizes a series of reporter gene cell lines [95]. The assay assessed biomarkers of DNA damage, protein misfolding, as well as oxidative and cellular stress. There were significant reductions in oxidative stress and cytotoxicity for the HTP when compared to the 1R6F Kentucky reference cigarette, with the authors noting that the HTP showed “qualitative reductions in toxicological endpoints over the cigarette." The evidence discussed in this section with respect to commercial assays employing multiple cell lines indicates that test materials derived from HTPs display less pronounced *in vitro* toxicological effects when compared to test materials derived from cigarettes.

Summary of In Vitro Toxicology Data

The results discussed in this section of the review demonstrate that HTPs elicit *in vitro* toxicological effects in a range of cell lines and assays when compared to negative controls, irrespective of the test material analyzed. However, the observed in vitro toxicological effects for HTPs are typically reported to be significantly less than those observed either with commercially available cigarettes or scientific reference cigarettes. With respect to the experimental methodologies employed, no whole smoke/aerosol exposure studies were identified that utilized cell lines derived from either the oral cavity or cardiovascular system, indicating a gap in the literature. The greatest evidence base was present for cell lines derived from the respiratory tract, for which all three test materials had been investigated. As noted in the original review [11], extracts of HTP aerosol have consistently been shown to have decreased (or absent) mutagenic, genotoxic, and cytotoxic effects when compared to cigarette smoke extracts. Nevertheless, the strongest evidence for the assessment of HTPs is likely to be derived from actual health outcomes in cohorts of HTP users compared to cohorts of smokers and non-smokers.

*In vivo *toxicology

This section of the review will discuss articles that have reported the *in vivo* toxicological effects of HTPs. Imperial Brands Plc does not test on animals unless required by regulatory authorities. Therefore, this section of the review solely discusses *in vivo* studies conducted by other researchers.

A total of twelve articles were identified that detailed an *in vivo* assessment of the effects of exposure to HTPs [66,70,96-105]. Eleven articles provided novel data [66,70,96,98-105] while one study provided a secondary analysis of data from a previous study [97]. All identified studies investigated the *in vivo* effects of exposure to HTPs in either mice or rats. Those studies that did not directly compare the effects of exposure to HTP aerosol with exposure to mainstream cigarette smoke were excluded from further discussion [66,96,98,99,104]. 

Cardiovascular System

In the first study, nine- to 11-week-old Sprague Dawley rats were exposed to HTP aerosol (IQOS) or mainstream cigarette smoke (Marlboro Red) while anesthetized using a nosecone [100]. Each product was puffed a total of 10 times, using a 30-second repeating cycle over five minutes (a five-second puff followed by 25 seconds of exposure to air). Both products were puffed using the International Organization for Standardization (ISO) puffing regimen. Endothelial function was assessed through the quantification of arterial flow-mediated dilation after surgical occlusion of the common iliac artery. Flow-mediated dilation was significantly impaired by exposure to both HTP aerosol (11.2±2.2% pre-exposure and 5.2±3.2% post-exposure) and to mainstream cigarette smoke (9.0±3.3% pre-exposure and 3.2±2.3% post-exposure). No significant impairment was seen in the control group exposed only to filtered air (7.8±2.3% pre-exposure and 7.9±4.3% post-exposure; p=0.98). The extent of flow-mediated dilation impairment ranged between 40% and 60% but did not significantly differ between groups other than for the controls. The authors concluded that all products investigated “impair flow-mediated dilation after a single session comparably to combustible cigarettes." The results of this study are comparable to those previously reported by the same researchers, who found the same association between HTP aerosol and mainstream cigarette smoke exposure and flow-mediated dilation in anesthetized rats [106]. A separate study by the same researchers concluded that “there is no single constituent or class of constituents responsible for acute impairment of endothelial function by (cigarette) smoke exposure; rather, we propose that acute endothelial dysfunction by disparate inhaled products is caused by vagus nerve signaling initiated by airway irritation” (although this study did not directly investigate HTP aerosols as part of its experimental methodology) [107].

In the second study, eight- to 10-week-old Sprague-Dawley rats were exposed in a nose-only fashion to HTP aerosol (IQOS) or mainstream cigarette smoke (Marlboro Red) for five days per week for eight weeks. Each daily session involved exposure to 10 puffs taken over five minutes. Both products were puffed using the ISO puffing regimen [105]. Systolic blood pressure, cardiac function, and histochemistry were then quantified in the rats. Exposure to all non-air conditions altered systolic blood pressure significantly when compared on a pre- versus post-exposure basis. Mainstream cigarette smoke and HTP aerosol acted similarly in increasing systolic blood pressure (>10 mmHg) after the first exposure, with relatively moderate acute increases on subsequent days. Analysis of cardiac function using echocardiography indicated that exposure for eight weeks progressively reduced both ejection fraction and fractional area change in all non-air exposure groups compared to their baseline levels. Left ventricular end-systolic volume and left ventricular end-diastolic volume were gradually increased in non-air exposure groups. At the end of the eight-week exposure period, left ventricular end-systolic volume and left ventricular end-diastolic volume were enlarged significantly in all non-air exposure groups compared to baseline values (p<0.05), along with an increase in left ventricular mass compared to baseline and to the air group (p<0.001). The authors concluded that exposure to HTP aerosol or mainstream cigarette smoke “results in an increased susceptibility to atrial fibrillation and ventricular tachycardia with reduced heart rate variability, cardiac sympathetic hyperinnervation, interstitial fibrosis, and electrophysiological changes in otherwise healthy rats." The available experimental data in relation to the cardiovascular system suggest that HTP aerosol may produce comparable effects on cardiovascular-related endpoints to those observed with mainstream cigarette smoke.

Respiratory System

In the first study, 12-week-old male mice were exposed to HTP aerosol (IQOS; Marlboro heatstick regular) or mainstream cigarette smoke (Peace non-filtered cigarettes) [101]. Mice were exposed for 30 minutes per day, five days a week, for six months. Six 15-ml puffs were taken per minute for each product, and the resultant aerosol or smoke was diluted to a 3.5% dilution with compressed air prior to exposure. The endpoints investigated were related to pulmonary morphology and function: histopathological assessment of quantitative endpoints for emphysema (mean linear intercept (MLI) and destructive index (DI)) and pulmonary function parameters (inspiratory capacity (IC), static compliance (Cst), static elastance (Est), and lung airway resistance (Rn)). Exposure to HTP aerosol was reported to produce pulmonary emphysema in mice similar to that observed with exposure to mainstream cigarette smoke. Airspace enlargement, quantified by the determination of the MLI, was significantly increased after exposure to both HTP aerosol and mainstream cigarette smoke when compared to controls. Alveolar wall destruction, quantified by the determination of the DI, was significantly increased after exposure to both HTP aerosol and mainstream cigarette smoke when compared to controls. Exposure to cigarette smoke, but not HTP aerosol, significantly increased total counts of cells present in bronchoalveolar lavage fluid (BALF), a traditional marker of inflammation, compared to controls. Exposure to HTP aerosol produced significantly increased levels of neutrophils and lymphocytes, but not macrophage levels, compared to controls. Exposure to cigarette smoke produced significantly increased levels of neutrophils and lymphocytes, but not macrophage levels, compared to controls. Exposure to cigarette smoke, but not to HTP aerosol, increased IC and Cst and decreased Est compared to controls. No changes in Rn were observed for either exposure group compared to controls. The statistical analysis reported in the study compared exposures with controls; there is no indication that any form of statistical analysis was conducted to investigate the observed differences in results produced by the two exposure groups. The authors concluded that “long-term exposure to IQOS aerosol induced emphysema in murine lungs via apoptosis. HTPs are, thus, not safer compared with conventional cigarettes and work in ways different from conventional cigarettes.”

In the second study, eight-week-old male mice (n=24) were whole-body exposed to HTP aerosol (IQOS; n=8), mainstream cigarette smoke (Marlboro Red; n=8) or filtered air (n=8) twice per day, five days per week, for 24 weeks [102]. Each exposure session lasted 30 minutes, with five heatsticks and five conventional cigarettes being used (12 puffs taken per heatstick and eight puffs taken per Marlboro Red cigarette). Both products were puffed using the Health Canada Intense Puffing regimen. The endpoints investigated were related to pulmonary morphology and function tests: histopathological assessment of quantitative endpoints for emphysema, including MLI and pulmonary function parameters, and oxidative stress (inflammatory cytokine secretion in serum, bronchoalveolar lavage fluid (BALF), and lung tissue homogenate). Exposure to HTP aerosol and mainstream cigarette smoke produced statistically significant alterations in seven of the 10 pulmonary function parameters quantified (respiratory system resistance (Rrs); Cst; respiratory system compliance (Crs); lung airway resistance (Rn); tissue damping (G); tissue elastance (H); forced expiratory volume per second vital capacity at 50 ms (FEV0.05/FVC)), with no statistically significant difference between the effects of the two exposure groups indicating comparable effects. Exposure to HTP aerosol produced less profound alterations in the remaining three pulmonary function parameters (forced vital capacity (FVC), inspiratory capacity (IC), and peak expiratory flow (PEF)) than exposure to mainstream cigarette smoke, with statistically significant differences between all three exposure groups. Exposure to HTP aerosol produced histological changes in lung tissue consistent with pulmonary emphysema in mice, similar to those observed with exposure to mainstream cigarette smoke. Airspace enlargement, quantified by the determination of the MLI, was significantly increased after exposure to both HTP aerosol and mainstream cigarette smoke when compared to controls, and no statistically significant differences were seen between the two exposure groups. A comparable relationship was observed for collagen deposition in tissues surrounding the airways. Levels of IL-6 and TNF-⍺ were significantly elevated by exposure to both HTP aerosol and mainstream cigarette smoke in all three matrices investigated, with no statistically significant differences between exposure groups. Levels of IL-1β, IFN-γ, and IL-8 in all three matrices were not significantly altered in any of the exposure groups. The authors concluded that “chronic exposure to IQOS aerosol results in impaired pulmonary function and lung tissue damage; hence, the concern should be raised regarding the long-term safety of this product.”

In the third study, eight-week-old male mice were split into equal groups and exposed in a nose-only fashion exclusively to HTP aerosol (IQOS), exclusively to mainstream cigarette smoke (3R4F Kentucky reference cigarette), or both as an experimental surrogate of dual product use [103]. Exposure occurred during two three-hour exposure sessions (split for dual product use groups) for seven consecutive days. The endpoints investigated were related to lung injury markers (wet-to-dry ratio, albumin concentration in BALF, expression of IL-1β, IL-6, and TNF-⍺, histopathology examination, reactive oxygen species (ROS) production, and assessment of cellular apoptosis). Exclusive exposure to HTP aerosol did not produce statistically significant differences in wet-to-dry ratio, albumin concentrations in BALF, expression of IL-1β, IL-6, and TNF-⍺, ROS production, or percentage of apoptotic cells when compared to controls exposed to filtered air. Those mice exclusively exposed to mainstream cigarette smoke or dual product use combinations demonstrated statistically significant differences in albumin concentrations in BALF, expression of IL-1β, IL-6, and TNF-⍺, ROS production, or percentage of apoptotic cells when compared to controls exposed to filtered air. Only those animals exposed exclusively to mainstream cigarette smoke demonstrated statistically significant increases in the wet-to-dry ratio. The statistical analysis reported in the study compared exposures with controls; there is no indication that any form of statistical analysis was conducted to investigate the observed differences in results produced between exposure groups. The authors concluded that “substituting 50% of daily cigarette smoke exposure (with) HTP aerosol did not result in significant attenuation of acute lung injury.”

In addition to articles providing novel data, a single article was identified [97], which reported a secondary analysis of data obtained from an 18-month rodent inhalation study conducted using the A/J mouse [108]. The inhalation study was undertaken to investigate the effect of lifetime exposure to HTP aerosols compared with that of exposure to mainstream smoke from the 3R4F Kentucky reference cigarette on toxicity and carcinogenicity endpoints. In this study, non-exposed A/J mice spontaneously developed lung tumors with age, and the authors therefore deemed it necessary to determine a method to discriminate spontaneous lung tumors from those resulting from smoke/aerosol exposure, as the tumors were indistinguishable by histological assessment. The researchers developed a 13-gene signature based on their data and data from a separate A/J mouse mainstream cigarette smoke exposure study [109]. Application of this gene signature to data from the 18-month lifetime exposure study resulted in the separation of mainstream cigarette smoke-induced lung tumors from spontaneous lung tumors observed in non-exposed mice. Lung tumors from mice exposed to HTP aerosol were significantly different from those observed in mainstream cigarette smoke-exposed mice but not significantly different from spontaneous tumors observed in non-exposed mice. The authors concluded that “tumor classification using this gene signature demonstrated a significant dissimilarity between lung tumors from 3R4F cigarette smoke-exposed and sham mice. The same signature also highlighted a significant dissimilarity between lung tumors from THS 2.2 aerosol- and 3R4F cigarette smoke-exposed mice, suggesting a different effect for the two exposures." The available experimental data in relation to the respiratory system suggests that HTP aerosol may produce less pronounced effects on respiratory-related endpoints than those observed with mainstream cigarette smoke.

Immune System

A single article was identified that investigated the effect of HTP aerosol and mainstream cigarette smoke on localized and systemic symptoms of antigen-induced arthritis [70]. Eight-week-old male mice were exposed to HTP aerosol from IQOS (Marlboro heatstick consumables) or mainstream smoke from Marlboro Red cigarettes. For each hour of exposure, 12 cigarettes and 24 heatsticks were used. Both products are puffed using the Health Canada Intense Puffing regimen. Animals received repeated injections of methylated bovine serum albumin into their knee joints to induce antigen-induced arthritis. Animals were then exposed for one hour twice per day for seven consecutive days after the second immunization (between days 14 and 20 after the first immunization). Exposure to mainstream cigarette smoke, but not HTP aerosol, worsened symptoms of antigen-induced arthritis. No statistically significant differences in four of the five symptoms of antigen-induced arthritis (mechanical hyperalgesia, oedema, neutrophil-extracellular traps, and MCP-1 levels) were observed for mice exposed to HTP aerosol when compared to air-exposed controls. With respect to synovial fluid cellularity, quantified levels after HTP aerosol were significantly decreased when compared to air controls and those mice exposed to mainstream cigarette smoke. Exposure to HTP aerosol led to low-grade lung inflammation similar to airflow-exposed mice but higher than the naïve group (who did not receive any vaccinations). The authors noted that “heat-not-burn tobacco exposure following a controlled Health Canada Intense Smoking regime does not cause prominent lung inflammation, as has been claimed by the literature." The available experimental data in relation to the immune system suggests that HTP aerosol may produce less pronounced effects in an experimental model of antigen-induced arthritis when compared with those observed with mainstream cigarette smoke. However, additional data is required to support this initial study.

Summary of In Vivo Toxicology Data

The available evidence suggests that exposure to HTP aerosols yields significantly reduced, or comparable, biological effects in rodents when compared to those observed with cigarette smoke in a range of tissues. This conclusion is consistent with that reported in the original review and therefore remains valid.

However, several limitations of the identified *in vivo* studies should be noted. All of the studies identified investigated the same HTP. As such, the findings cannot be extrapolated to other commercially available HTPs, and, as there was no comparator assessed, it is not possible to assess whether differences would be observed with different HTPs. All of the identified studies, which provided novel data, investigated a single dose level. This precludes any assessment of a potential dose-response relationship, and further information is required to determine whether the single dose levels used are representative of those associated with human exposure. Rodents are obligate nasal breathers, meaning that they can only breathe through their nose. As a result, the processes by which rodents inhale aerosols will be fundamentally different than those observed in human respiratory physiology. The anatomical structure of the rodent respiratory tract in rodents is fundamentally different from that of humans and therefore will lead to meaningfully different exposure and retention patterns.

Overall, the relevance of *in vivo *studies for assessing the effects of HTP aerosols on human exposure is unclear given their inherent limitations. The strongest evidence for the assessment of HTPs is likely to be derived from actual health outcomes in cohorts of HTP users compared to cohorts of smokers and non-smokers.

Biomarkers

This section of the review will detail those studies that have quantified or discussed levels of biomarkers of exposure (BOE) and/or biomarkers of potential harm (BOPH) associated with HTP use. These studies will include those conducted under clinical confinement conditions and with real-world users.

Exhaled Carbon Monoxide and Salivary Cotinine

Two studies utilizing data from larger cohorts of product users were identified that detailed the level of exhaled carbon monoxide (eCO) and/or salivary cotinine in HTP users in comparison to other product use groups. The first study investigated exhaled carbon monoxide (eCO) and salivary cotinine levels in current users of cigarettes, HTPs, and/or EVPs [110]. Current users from a household survey and a survey conducted at informal outdoor smoking locations where smokers typically congregated were classified into seven product use groups: exclusive users of each product type (three groups: cigarette smokers, HTP users, and EVP users), dual users for each possible product type combination (three groups: cigarettes and HTPs, cigarettes and EVPs, and HTPs and EVPs), and triple product users. eCO and salivary cotinine levels were quantified for a subset of the survey participants. Compared with exclusive cigarette smokers, all other product use groups demonstrated significantly lower levels of eCO. The median salivary cotinine level was equivalent to 100 to 200 ng/ml in exclusive cigarette smokers and exclusive HTP users and was equivalent to 200 to 500 ng/ml in dual product users using HTPs and triple product users, with no statistically significant differences between these values. The authors concluded that “this study showed for the first time that exclusive HTP users had a lower eCO level than cigarette users (either used exclusively or concurrently with HTPs), but saliva cotinine levels were similar between the three groups in a real-world setting." It should be noted, however, that the authors did not include a group of never-users in their study design.

The second study investigated levels of eCO in a cross-sectional analysis of nicotine- and tobacco-containing product users and non-product users in Kuala Lumpur, Malaysia [111]. Of the participants (n=657), 52.1% were non-product users. With respect to product users, 48.3% were exclusive cigarette smokers, 27.3% were multiple product users, 20.9% were exclusive EVP users, and 3.5% were exclusive HTP users. Median eCO levels were 13 ppm, 7 ppm, 2 ppm, 2 ppm, and 1 ppm for exclusive cigarette smokers, multiple product users, exclusive EVP users, exclusive HTP users, and non-product users, respectively, with statistically significant differences between all groups. The authors concluded that “the findings suggest that electronic cigarettes and HTP users are exhaling less carbon monoxide.”

Exhaled Breath Analysis

A single study identified that quantified levels of ethylene present in exhaled breath samples after controlled use of cigarettes, EVPs, and HTPs [112]. Seven current smokers were recruited into the study, and they used one of the products each day for a total of three days after a 12-hour period of abstinence. Each period of product use consisted of two sessions of four minutes, separated by one minute. The baseline mean level of ethylene present in the exhaled breath of the study participants prior to product use was 26.7 ppb. The use of cigarettes produced a mean exhaled breath ethylene concentration of 687 ppb, while EVP and HTP use produced levels of 56 ppb and 48 ppb, respectively, equivalent to decreases of approximately 92% and 93%.

Forced Switching Studies

Two switching studies were identified that investigated the effects of controlled product use under conditions of clinical confinement on BOE. In the first study, the effects of product switching among adult Japanese smokers were assessed in clinical settings over a five-day period to investigate exposure to smoke toxicants [113]. Smokers (n=89) were randomly assigned to one of six study groups. Four groups were asked to switch to using one of three separate commercially available eHTPs or one aHTP; one group continued to smoke their usual brand of cigarette; and the final group stopped smoking and abstained from using nicotine- and/or tobacco-containing products. The three eHTPs investigated were IQOS 3 Multi (used with Marlboro heatstick regular), Glo Pro (used with Kent Neo Sticks Bright Tobacco), and Ploom S (used with MEVIUS Regular Taste). Fifteen BOEs (14 HPHCs and pyrene) were quantified at baseline, day three, and day five in 24-hour urine samples and exhaled breath. Under conditions of clinical confinement, product use was monitored to ensure compliance with the study design and protocol. On day five, relative to the group who continued to smoke, significant reductions in all BOE were observed in study participants who switched to the three HTPs, to the hybrid device, and in those study participants who stopped smoking. The authors concluded that “the results obtained in the present study indicate that switching (to the investigational products) was associated with significant reductions in exposure to most of the selected HPHCs relative to smoking continuation in Japanese smokers. Significantly, the magnitude of the reduction in exposure to most of the selected HPHCs observed in the HTP groups was close to that observed in the SS (stopped smoking) group.”

The study protocol for the second study, as described in the original review [11], detailed a controlled, single-center study involving 60 healthy subjects, divided into six product use groups (five nicotine product user groups and one non-user group) based on the sole use of their products of choice (cigarettes, HTPs, EVPs, oral tobacco products, and oral/transdermal nicotine replacement therapy (NRT) products) [114]. The results of the study have been published in several articles, with each article looking at different biomarker classes [115-121]. The subjects were confined in a clinical setting for a period of 76 hours, during which time unrestricted use of their product of choice was permitted as described (cigarettes, HTPs, EVPs, oral tobacco products, and oral/transdermal nicotine replacement therapy products). The aim of the study was to identify biomarkers and/or biomarker patterns in blood or urine, which may be used to distinguish between product categories as a means to determine compliance in long-term clinical studies. The mean estimated nicotine intake for HTP users was 4.49 mg per day, while the estimates for cigarette smokers, EVP users, nicotine replacement therapy users, and oral tobacco users were 6.17 mg per day, 9.38 mg per day, 7.70 mg per day, and 17.31 mg per day, respectively [115].

The exposure of HTP users to tobacco-specific nitrosamines (TSNAs), minor alkaloids, polycyclic aromatic hydrocarbons (PAHs), aromatic amines, ethylene oxide, 1,3-butadiene, and benzene was reported to be at levels comparable to those observed for non-users [116-120]. Overall, the authors concluded that unique differentiation between product use groups by means of a single biomarker was not possible for users of oral tobacco products, HTP users, and nicotine gum users [121]. Large differences in terms of exposure levels were observed between cigarette smokers and all other product use groups. In contrast, differences were less pronounced between the four groups of non-cigarette users (HTP users, EVP users, oral tobacco users, and nicotine gum users) and non-users.

A systematic review was identified that aimed to detail BOE associated with bladder cancer and directly compare the levels quantified in the urine of HTP users (both eHTP and cHTP) with levels quantified in the urine of cigarette smokers [122]. The review of the scientific literature was conducted in December 2020. Biomarkers and their parent compounds were classified according to International Agency for Research on Cancer (IARC) monographs and cross-referenced with the Collaborative on Health and the Environment Toxicant and Disease Database to determine associations with bladder cancer. A total of eleven studies met the authors’ inclusion criteria. A total of 21 BOEs present in the urine of HTP users, which reflected exposure to 21 unique parent compounds, were identified in the studies.

The authors of the systematic review reported that HTP users generally had significantly lower BOE levels compared to cigarette smokers in each study, though levels of nicotine, as measured by nicotine equivalents, were comparable between HTP users and cigarette smokers within each study [122]. The mean levels of NNAL (the primary metabolite of NNK) quantified in HTP users were 36.6% to 76.% lower than those quantified in cigarette smokers. NNN levels were 42.1% to 93.1% lower, 2-NA levels were 81.2% to 90.3% lower, 4-ABP levels were 60.0% to 85.1% lower, 3-OH-BaP (a metabolite of BaP) levels were 57.1% to 72.4% lower, BaP levels were 47.2% to 68.8% lower, 1-OHP (the primary metabolite of pyrene) levels were 28.1% to 71.0% lower, and o-toluidine levels were 44.5% to 71.9% lower across all relevant studies. No quantitative data was provided for fluorene, naphthalene, or phenanthrene in the paper.

No urinary BOE for known bladder carcinogens was reported to be found at higher levels in HTP users compared to cigarette smokers in any of the studies identified and reviewed. The authors concluded that “the level of carcinogen exposure in HTP users appears to be lower than that of combustible cigarette smokers but significantly higher than that of never-smokers." It should be noted, however, that five of the eleven studies identified by the authors and included in their analyses reported data relating to cHTPs rather than eHTPs. An earlier review by the same authors on the potential role of HTPs in urologic health noted that while biomarkers of exposure to bladder carcinogens had been observed in the urine of HTP users, “whether or not the levels of exposure are high enough to contribute to human carcinogenesis remains to be determined” [123]. The available data suggests that HTP use is associated with reduced exposure to chemical toxicants found in cigarette smoke, as assessed using BOE when compared with cigarette use.

A ‘Real-World’ 360-Day Ambulatory Study

An ambulatory clinical study aimed at measuring levels of BOE and BOPH is underway in the UK. Cigarette smokers have been randomized to continue to smoke cigarettes as usual, switch to exclusive use of the Glo HTP (also described as THP1.1 (RT) in the scientific literature), or quit tobacco product use. A group of never-smokers was also recruited for comparative measures of BOE and BOPH. The study design, protocol, and statistical plan have been published separately [124,125]. Furthermore, articles providing results in relation to the intermediate 90-day [126] and 180-day time points [127] have been previously published (and discussed in detail in the original review [11]). Results from these intermediate time-points reported sustained reductions in the majority of BOE compared to baseline at both time-points for both the group of cigarette smokers randomized to use of the Glo HTP and the group randomized to quit tobacco product use. At 180 days, several BOPH, including 8-epi-prostaglandin F2⍺ type III (8-epi-PGF2⍺), white blood cell count, and fractional concentration of exhaled nitric oxide, had changed in a favorable direction (towards those seen in never-smokers) for both of these groups, while levels in those participants randomized to continued cigarette use either remained constant or changed in an unfavorable direction (away from those observed in never-smokers). No data in relation to BOPH was provided for the 90-day time period.

Gale et al. provided an update to this study, presenting data from the 360-day time point [128]. This data indicated a substantial and sustained reduction in BOE levels for both study participants randomized to exclusive HTP use and participants randomized to quit tobacco use. Many of the observed reductions were of a similar order of magnitude for both groups. Percentage reductions in non-nicotine-related BOE for participants randomized to exclusive HTP use compared to baseline ranged between 26% for total NNN and 96% for 2-cyanoethylmercapturic acid (CEMA). Total nicotine equivalents showed a percentage reduction of 29%. Several BOPH were reported to have changed in a favorable direction over the 360-day period for participants randomized to exclusive HTP use and participants randomized to quit tobacco use. These include 8-epi-PGF2⍺, 11-dehydrothromboxane B2 (11-dTx B2), concentration of fractionated exhaled nitric oxide (FeNO), white blood cell count, and soluble intercellular adhesion molecule (sICAM-1). In those study participants randomized to continued use of cigarettes, several BOPHs were reported to have moved in an unfavorable direction (FeNO, white blood cell count, sICAM-1, and FEV1). The authors concluded that “our findings, alongside chemical and toxicological studies undertaken on the tobacco heating product (THP) used in this study, lead to the conclusion that smokers who would have otherwise continued to smoke and instead switch entirely to the use of this THP will reduce their exposure to tobacco smoke constituents and, as a consequence, are reasonably likely to reduce disease risks compared to those continuing to smoke.”

Summary of Biomarkers Data

Concerning BOE, the results discussed in this section of the review clearly demonstrated that the use of HTPs in both clinical and ‘real-world’ settings is associated with a statistically significant reduction in levels of non-nicotine-related BOE to select toxicants associated with cigarette smoking. This conclusion is consistent with that reported in the original review, which remains valid.

With respect to BOPH, results from the 360-day time-point of an ambulatory clinical study discussed in this section of the review demonstrated that several biomarkers of potential harm moved in a favorable direction (towards those seen in never-smokers) for study participants randomized to both HTP use and to product abstinence, while levels moved in non-favorable directions for those study participants who continued to smoke cigarettes. This provides additional data beyond that reported in the original review.

Nicotine pharmacokinetics and abuse liability

This section of the review will detail those studies that have investigated the pharmacokinetic profile of nicotine and the abuse liability profile of HTPs under controlled clinical conditions. An additional article was identified that discussed the potential effects of switching to HTPs from cigarettes on the metabolism of phenytoin, an anti-epileptic medication whose metabolism was reported to be increased by smoking [129]. However, no quantitative data on its metabolism after HTP use was reported, and therefore the article will not be discussed further in the update.

Pharmacokinetic Profile of Nicotine Delivery Associated With Heated Tobacco Product Use Under Controlled Conditions

Five controlled clinical studies involving adult smokers were identified that have attempted to estimate the pharmacokinetic profile of nicotine delivery from HTPs and compare this delivery with that observed with cigarettes and/or EVPs. In these studies, three key pharmacokinetic parameters were typically quantified for each product investigated: C_max_ (the maximum plasma concentration of nicotine produced over the sampling period), T_max_ (the time at which C_max_ occurred), and AUC_0-t_ (a measure of overall nicotine exposure over the sampling period). An increase in AUC_0-t_ and C_max_ values, as well as a shorter T_max_ value, for the investigative product(s) when compared to cigarette use, would suggest an increase in abuse liability. An additional article was identified that reported C_max_ and T_max_ values for IQOS use under controlled conditions [130]. However, the study did not include a comparator group involving the use of cigarettes, and therefore the study will not be discussed further in this section.

In the first study, 40 adult smokers participated in a five-arm, randomized crossover clinical confinement study that investigated three variants of the JUUL EVP in addition to the IQOS HTP and participants’ usual brand of cigarettes [131]. Each product was investigated following a ten-minute period of* ad libitum* use and, at least six hours later, after a five-minute period of controlled use in which a total of ten puffs were taken from each product. The HTP with Birch heatsticks (18 mg/g nicotine) was compared to three JUUL e-liquid variants assessed (18 mg/ml, 40 mg/ml, and 59 mg/ml nicotine contents, respectively). Plasma nicotine concentrations were determined for periods of 90 and 60 minutes following the *ad libitum* and controlled use sessions, respectively. Pharmacokinetic parameters for these products for both product use conditions are shown in Table 2. Baseline-adjusted C_max_ and AUC_0-t_ values for participants’ usual brand of cigarettes and the comparable EVP variant were significantly higher than those determined for the HTP, while T_max_ showed no difference between the EVP and the HTP, indicating that the HTP produced a lower maximum nicotine concentration and lower overall nicotine exposure while producing a comparable time to maximum nicotine concentration. The comparable EVP variant was rated higher by study participants than the EVP on subjective measures associated with switching away from cigarettes and was generally rated as more satisfying and effective at reducing cravings, with the authors noting that “JUUL products were significantly more satisfying and effective at reducing cravings than the HTP.”

**Table 2:** Pharmacokinetic parameters determined for HTP use ^1^Mean values reported for the study of Goldenson et al. and McDermott et al.; median values reported for the study of Hardie et al. and Rabenstein et al. ^2^AUC0-t, a measure of total nicotine exposure over the entire measurement period; t=90 minutes after ad libitum product use and t=60 minutes after controlled product use in the study of Goldenson et al.; t=240 minutes in studies of Hardie et al. and McDermott et al.; t=90 minutes in the study of Rabenstein et al.; t=30 minutes in the study of Vukas et al. ^3^Units of ng*min/ml were used by studies of Goldenson et al., Hardie et al., and McDermott et al.; units of ng/ml*h were used by the study of Rabenstein et al. Conversion of results reported by Rabenstein et al. from ng/ml*h to ng*min/ml was not conducted as the study design was significantly different from that of the other three studies and the AUC0-t parameter allowed direct comparison between overall nicotine exposure between product use groups within the same study. HTP: heated tobacco products; NRT: nicotine replacement therapy

Study	Products Investigated (And Their Nicotine Content Where Provided)	Mean Maximum Plasma Nicotine Concentration (C_max_; ng/ml)	Mean/Median^1^ Time to Maximum Plasma Nicotine Concentration (T_max_; minutes)	Area Under the Curve^2,3^ (AUC_0-t_)
Goldenson et al. [131] (ad libitum product use)	Participants’ usual brand (CC)	CC: 31.66	CC: 12.51	CC: 1,472
	IQOS (18mg/ml)	IQOS: 18.22	IQOS: 15.53	IQOS: 893
	JUUL (18mg/ml)	JUUL: 13.98	JUUL: 12.15	JUUL: 687
Goldenson et al. [131] (controlled product use)	Participants’ usual brand (CC)	CC: 24.83	CC: 9.52	CC: 726
	IQOS (18mg/ml)	IQOS: 13.68	IQOS: 6.98	IQOS: 433
	JUUL (18mg/ml)	JUUL: 8.71	JUUL: 6.56	JUUL: 250
Hardie et al. [132]	Participants’ usual brand (CC)	CC: 22.7	CC: 6.03	CC: 1,317
	Glo variant 1	Glo variant 1: 8.6	Glo variant 1: 4.05	Glo variant 1: 519
	Glo variant 2	Glo variant 2: 10.5	Glo variant 2: 4.07	Glo variant 2: 671
	NRT inhaler	NRT inhaler: 2.3	NRT inhaler: 15.03	NRT inhaler: 333
Rabenstein et al. [133]	Participants’ usual brand or Marlboro Red (CC)	CC: 24.0	CC: 75	CC: 24.8
	IQOS (4.7-5.1mg per consumable)	IQOS: 17.7	IQOS: 75	IQOS: 17.3
	JUUL (18mg/ml)	JUUL: 8.0	JUUL: 90	JUUL: 8.3
Vukas et al. [134]	Marlboro Red (CC)	Marlboro Red: 25.1	Marlboro Red: 6	Marlboro Red: 6.1
	IQOS 3 Duo (Amber HEETS)	IQOS 3 Duo: 14.9	IQOS 3 Duo: 6	IQOS 3 Duo: 4.0
	Glo (Neo Tobacco Bright sticks)	Glo: 11.6	Glo: 6	Glo: 3.0
McDermott et al. [135]	Participants’ usual brand (CC)	CC: 21.7	CC: 9.1	CC: 1,513
	Pulze (three consumable variants; intense American blend, regular American blend, regular menthol)	Pulze (intense American): 10.5	Pulze (intense American): 7.5	Pulze (intense American): 746
		Pulze (regular American): 7.5	Pulze (regular American): 11.7	Pulze (regular American): 586
		Pulze (regular menthol): 8.2	Pulze (regular menthol): 8.7	Pulze (regular menthol): 586

In the second study, thirty-two adult smokers participated in a four-arm, randomized, crossover clinical confinement study that investigated two variants of the Glo HTP (differing in their nicotine content), participants’ usual brand of cigarettes, and a 15 mg Nicorette NRT inhaler [132]. Each product was used *ad libitum* for a five-minute period in each of the four study periods, which were conducted on separate days. Blood samples were taken to determine the pharmacokinetic profile of nicotine delivery, and questionnaires were administered to assess subjective effects related to product liking, overall intent to use again, urge for product, and urge to smoke. Pharmacokinetic parameters are shown in Table 2. C_max_ was higher for the participants’ usual brand of cigarettes (22.7 ng/ml) when compared to either variant of the HTP (8.6 and 10.5 ng/ml respectively) and the NRT inhaler (2.3 ng/ml). Median T_max_ was significantly longer for the NRT inhaler (15.03 minutes) than for any of the tobacco-containing products (4.05 to 6.03 minutes). Subjective scoring relating to product liking and overall intent to use was highest for the participants’ usual brand of cigarettes and higher for both variants of the HTP compared to the NRT inhaler. The urge to smoke was reduced more by the use of participants’ usual brand of cigarette to a greater degree than by the use of any of the other study products. The authors concluded that “the abuse liability of the tobacco heating products lies between that of the subjects usual brand cigarettes and the NRT” and that “since tobacco heating products have lower emissions and reduced* in vitro* biological activity compared to cigarettes, and since switching to using tobacco heating products reduces exposure to harmful toxicants, the presence of some degree of abuse liability of the tobacco heating product supports tobacco harm reduction such that it may provide an appealing and accepting alternative to cigarette smoking in adult smokers and be supportive of their switching away from harmful cigarette smoking.”

In the third study, a pod-based EVP, the IQOS HTP, and cigarettes were each used *ad libitum* by fifteen experienced users of each product for a 90-minute period [133]. With respect to the cigarettes used in the study, participants could choose from their usual brand, which they provided themselves, or Marlboro Red which was provided to them by the researchers. Pharmacokinetic parameters are shown in Table 2. Nicotine delivery was highest after cigarette use, followed by HTP use and EVP use. There were no statistically significant differences in any of the three quantified pharmacokinetic parameters between cigarette use and HTP use. When pharmacokinetic parameters were quantified after defined product use involving a total of ten puffs, as opposed to after *ad libitum* use, and with an additional HTP included (glo), no statistically significant differences were observed between the two HTPs for C_max_, T_max_ or AUC_0-30_ whilst statistically significant differences were observed between the HTPs and cigarette for C_max_ and AUC_0-30_ (both parameters being lower with HTPs compared to cigarettes) but not for T_max_ [134].

The most recent study investigated the nicotine delivery and subjective effects associated with the use of the Pulze HTP combined with three separate consumables, with comparison to participants’ usual brand of cigarettes [135]. Pharmacokinetic parameters are shown in Table 2. C_max_ and AUC values were significantly lower after HTP use when compared to cigarette use, while no statistically significant differences were observed between products for T_max_. All study products reduced the urge to smoke, as reported by participants. Results for subjective effects were comparable between HTP consumable variants and lower than those reported for participants’ usual brand of cigarettes. Product evaluation scores for the HTP variants across ‘satisfaction’, ‘psychological reward', and ‘relief’ were similar and lower than those associated with cigarette use. The authors concluded that “the Pulze heated tobacco system effectively delivers nicotine and generates positive subjective effects, including satisfaction and a reduced urge to smoke. This supports the conclusion that the Pulze heated tobacco system may be an acceptable alternative to cigarettes for adult smokers while having a lower abuse liability than cigarettes.”

Summary of Pharmacokinetic Data

A key requisite for any HTP and its impact on tobacco harm reduction is that it provides a satisfying alternative to adult smokers who would otherwise continue to smoke. An HTP with a comparable nicotine uptake profile to cigarettes is likely to be more satisfying to adult smokers considering HTPs as an alternative to continued cigarette smoking. The available pharmacokinetic data indicates that the use of HTPs provides a comparable nicotine pharmacokinetic profile to that observed with cigarette use, with decreased overall exposure to nicotine over the periods of assessment as quantified by the AUC parameter. Subjective assessments of abuse liability determined through the use of both validated questionnaires and PK endpoints indicate that HTPs have a decreased potential for abuse liability when compared to cigarettes, which would be of benefit in assisting smokers to transition away from continued cigarette use. These conclusions are consistent with those reported in the original review and, therefore, remain valid. Additional research is warranted to determine if differences exist between quantified pharmacokinetic values and whether product use is controlled or whether participants are free to use products as they wish.

Health effects

This section of the review will discuss those articles that have investigated or discussed the potential health effects associated with HTP use. These studies include a range of study designs, including epidemiological studies, clinical studies, and medical case reports.

Cardiovascular System: Epidemiological Studies

Three epidemiological studies were identified that assessed the effects of HTP use on cardiovascular endpoints. The first study aimed to assess the association between HTP use and high-density lipoprotein cholesterol (HDL-C) concentrations [136]. High HDL-C levels were reported by the authors to be favorable for health and to have been associated with a lower risk of heart disease. The study included a total of 48,771 participants who worked for six separate Japanese companies and who participated in the Japan Epidemiology Collaboration on Occupational Health (J-ECOH) Study, an ongoing multi-company study of workers. Under Japanese law, all workers are required to undergo a health assessment at least once a year, which includes a self-administered questionnaire, physical examinations, and laboratory examinations, including the analysis of blood biochemical parameters. As part of the J-ECOH study, participants were classified as never-smokers, former smokers, exclusive HTP users, exclusive cigarette smokers, or dual product users (of both HTPs and cigarettes) during their annual health assessment. Of the 48,771 participants, 82.5% were male with an average age of 45.5 years, with 9.3% reporting to be exclusive HTP users and 6.0% reporting to be dual product users. Exclusive HTP users were reported to have ‘modestly’, but significantly lower HDL-C levels when compared to never-smokers (who acted as the reference group), with the mean difference being -1.1 mg/dL (95% confidence intervals of -1.5 mg/dL to -0.6 mg/dL). Dual product users showed a greater difference (mean difference of -3.7 mg/dL; 95% confidence intervals of -4.2 mg/dL to -3.2 mg/dL), which was noted to be approximately comparable to that of exclusive cigarette smokers (mean difference of -4.3 mg/dL; 95% confidence intervals of -4.7 mg/dL to -3.9 mg/dL). There was no apparent gender effect in the reported association with product use and HDL-C concentrations. The authors concluded that “exclusive HTP users had lower HDL-C concentrations than never-smokers, although higher than exclusive cigarette smokers. Moreover, dual (product) users had HDL-C concentrations similar to those in exclusive cigarette smokers.” Further research is required to determine the clinical significance of these findings and whether they translate into changes in disease risk for product users.

The second study aimed to assess the association between HTP use and the incidence of prediabetes and diabetes [137]. The study investigated a separate cohort of participants (n=8,950) from the J-ECOH study that was previously investigated by the same group [136]. Diabetes and prediabetes were defined according to fasting blood glucose and HbA1c levels and self-reported diabetes treatment, using criteria defined by the American Diabetes Association. Exclusive HTP users were reported to have increased odds of prediabetes (odds ratio of 1.36; 95% confidence intervals of 1.25 to 1.47) and diabetes (odds ratio of 1.68; 95% confidence intervals of 1.46 to 1.94) than never-smokers. Dual product users had increased odds of prediabetes (odds ratio of 1.26; 95% confidence intervals of 1.13 to 1.39) and diabetes (odds ratio of 1.93; 95% confidence intervals of 1.63 to 2.29) than never-smokers. Exclusive cigarette smokers had increased odds of prediabetes (odds ratio of 1.12; 95% confidence intervals of 1.04 to 1.21) and diabetes (odds ratio of 1.57; 95% confidence intervals of 1.38 to 1.78) than never-smokers. The authors concluded that “HTP use was associated with an increased likelihood of prediabetes and diabetes” when compared to no product use, although the authors did not adjust for smoking history in their analysis. Furthermore, there is significant overlap across the 95% confidence interval ranges for each reported odds ratio, which suggests that the observed results may not be statistically meaningful.

The third epidemiological study used a cross-sectional design to investigate the relationship between HTP use, cigarette use, and white blood cell count [138]. The study used data from the Korean National Health and Nutrition Examination Survey and included a total of 9,747 separate data points from 3,952 men and 5,795 women. This cohort consisted of 6,490 non-smokers, 1,999 former smokers, 76 HTP users, and 1,182 cigarette smokers. White blood cell count was significantly increased in cigarette smokers, but there was no statistically significant effect reported for HTP users. However, it should be noted that there was a very small number of HTP users contained within the study (n=76; 0.78% of the total cohort), so the statistical power of the study may be limited. The currently available epidemiological evidence does not support the hypothesis that HTP use leads to an increase in cardiovascular risk.

Cardiovascular System: Clinical Studies

Five experimental clinical studies were identified that reported the effects of HTP use on cardiovascular endpoints. An additional study was identified but subsequently excluded from further analysis as its reported results in relation to heart rate variability were derived solely from a single study participant [139].

The first study quantified seven cardiovascular markers (heart rate, systolic and diastolic blood pressures, exhaled carbon monoxide, pulse wave velocity, augmentation index, and central systolic blood pressure) in 40 each of exclusive cigarette smokers, exclusive HTP users, and non-product users [140]. No information was provided regarding the product use history of the study participants. Both exclusive cigarette smokers and exclusive HTP users had a significantly higher augmentation index (a marker of endothelial function) (24.80±5.95% and 22.16±2.82%, respectively) when compared to non-product users (18.46±7.02%). No statistically significant difference was reported between the two product use groups. All of the other cardiovascular markers investigated were comparable between exclusive HTP users and non-product users at baseline. Cigarette smokers demonstrated a statistically significant elevation in exhaled carbon monoxide levels at baseline (8.00±3.86 ppm) when compared to both exclusive HTP users (1.15±0.37 ppm) and non-product users (1.11±0.56 ppm), with no statistically significant difference reported between exclusive HTP users and non-product users. Overall, the only cardiovascular endpoint that was meaningfully affected by HTP use was the augmentation index, with the observed increase in this endpoint being less pronounced than that observed with cigarette use.

The second study examined the effect of acute product use over a five-minute period on cardiovascular markers of arterial stiffness [141]. This crossover study included a total of 20 occasional smokers who used each of four products (a cigarette, a HTP (IQOS), a nicotine-containing EVP, and a nicotine-free EVP) over a five-minute period following a specific puffing regime for the EVP only. Study results indicated that the reactive hyperemia index (a marker of arterial stiffness, with the authors reporting that a decrease in this value is associated with acute damage to the endothelium) showed a statistically significant decrease after all product use scenarios, beginning fifteen minutes after use and persisting after sixty minutes, while the augmentation index increased significantly after all product use scenarios at both fifteen and sixty minutes after product use. The experimental methodology employed solely investigated an acute exposure scenario, and there is no indication that the study participants had any previous experience with HTPs prior to their involvement in the study. Nevertheless, the authors concluded that “direct comparison of a JUUL device with a HTP, a conventional cigarette, and a nicotine-free e-cigarette reveals a comparable effect of each device on peripheral hemodynamics, endothelial vasodilator function, and arterial stiffness.”

The third study investigated the role of gender in the cardiovascular effects of cigarette or HTP use [142]. The study comprised a gender-specific analysis of a previously published prospective observational study [143]. The study included 20 each of cigarette smokers, HTP users, all of whom were former cigarette smokers, and non-product users. Product users were required to have used their respective products for more than one month in order to be included in the study. Blood samples were collected for biochemical analyses, and blood pressure and flow-mediated dilation were quantified in all study participants. Bivariate and multivariate analyses indicated no statistically significant differences between male and female study participants when considered as an overall cohort or when stratified by the respective product use categories for any of the investigated markers (flow-mediated dilation, nitric oxide levels, sNox2-dp levels, H2O2 levels, sCD40L levels, sP-selectin levels, platelet aggregation, and cotinine levels). With respect to the observed differences between the respective product use groups, six of the eight endpoints (flow-mediate dilation, nitric oxide levels, H2O2 levels, sCD40L levels, platelet aggregation, and cotinine levels) were reported to not show statistically significant differences between cigarette smokers and HTP users, while sNox2-dp and sP-selection levels were reported to be present at lower levels in HTP users than in cigarette smokers. When compared to never-smokers, statistically significant differences were observed for all endpoints. The authors concluded that “smoking heat-not-burn cigarettes or traditional combustion cigarettes has a similar negative impact on reactive oxygen species generation and vascular reactivity, although heat-not-burn cigarette smokers display lower levels of markers related to platelet activation.”

In the fourth study, 160 healthy young adults, split into equal groups of cigarette smokers, HTP users, EVP users, and non-smokers, underwent an assessment of cardiovascular endpoints immediately before, immediately after, and 30 minutes after respective product use [144]. HTP users used a single consumable, cigarette smokers smoked a single cigarette, EVP users used an EVP for five minutes, and non-smokers simulated smoking for five minutes. HTP use had no statistically significant effect on blood oxygen saturation, heart rate, systolic blood pressure, or diastolic blood pressure when baseline levels were compared to those measured 30 minutes after product use. Comparisons between product use groups indicated no statistically significant differences in blood oxygen saturation, heart rate, systolic blood pressure, or diastolic blood pressure, although the spread of the data was significant, suggesting significant variability between participants.

The most recent study investigated clinical markers of endothelial dysfunction in 40 current users of either cigarettes or EVPs after controlled use of the IQOS HTP, EVP, and a cigarette [145]. The authors provided no indication as to how many of the study participants (n=40) had ever used an HTP prior to their involvement in the study but did note that those “smokers who were inexperienced in the use of e-cigarettes or HTPs were trained using e-cigarettes by an experienced e-cigarette user” and that “the use of HTPs was explained to the participants following the instructions made by an IQOS store." All study participants were required to take a minimum of one puff every thirty seconds for a total of ten puffs using each product. Use of the HTP as well as the different investigated EVPs produced exhaled carbon monoxide levels above those typically associated with non-smokers, while cigarette use increased levels significantly. Transient statistically significant increases in pulse wave velocity (the speed of the arterial pulse wave produced by contraction of the heart as it radiates out to the peripheral blood vessels) and the augmentation index were observed after HTP use. However, both returned to levels comparable to the baseline 45 minutes after product use and remained as such 60 minutes after product use. The authors concluded that “even a single consumption of a different nicotine delivery system or cigarette leads to a significant inflammatory reaction followed by endothelial dysfunction and increased arterial stiffness, causing cardiovascular disease." However, this study design is not reflective of real-world product use, as study participants followed a defined schedule for product use, and therefore the relevance of the findings is limited.

The currently available experimental data does not provide consistent evidence in relation to the acute cardiovascular effects associated with HTP use. The generalisability of the results obtained from acute exposure under controlled conditions to real-world product use may be limited.

Cardiovascular System: Review Articles

Several non-systematic reviews and a single systematic review relating to the potential cardiovascular effects of HTPs were identified. The first non-systematic review detailed the key findings from four studies in an attempt to detail the impact of HTP use on oxidative stress [146]. The authors concluded that “the safety profile of NGPs, including electronic cigarettes and tobacco heating products, seems to be higher than that of tobacco cigarettes, but further studies are needed to better understand the toxicological effects of these products with long-term exposure." The second non-systematic review noted that “there is limited data on the effects of heat-not-burn on general health and cardiovascular endpoints, a knowledge gap that warrants attention” [147]. The third non-systematic review detailed the key findings from two clinical trials, but the authors did not draw any firm conclusions of their own based on these results [148]. While the most recent non-systematic review detailed results from several experimental studies, included both in the original review [11] and this update, it did not draw any specific conclusions in regard to HTPs [149].

The single systematic review identified and assessed data from twenty-five articles published up to September 2022 [150]. Fifteen of the studies reported data relating to different variants of the IQOS HTP, while seven reported data relating to other HTPs (the final three articles related to cHTPs). The authors of the systematic review concluded that “data synthesized in this systematic review reveals that by switching to HNB tobacco products from either conventional cigarettes, e-cigarettes, or other types of tobacco products, the reduced exposure is linked to improvements in biomarkers of effect-related to cardiovascular disease risk, which carries clinical implications with respect to mechanistic pathways engaged in CVD development and progression." The currently available reviews relating to the cardiovascular effects of HTP use suggest a reduced likelihood of harm when compared to cigarette use. However, further research is warranted.

Respiratory System: Pulmonary Function Parameters

Three studies were identified that reported on the effects of HTP use on pulmonary function parameters in study participants without pre-existing pulmonary disease. A study protocol detailed in our original review [11] described a five-year single-center observational study that aimed to evaluate the frequency of exacerbations, respiratory symptoms, physical exercise intolerance, and abnormal lung functions in study participants who used the IQOS HTP when compared to those study participants who smoked cigarettes [151]. Results from the study, relating to one-, two-, and four-year time points and published across several articles, were identified [152-154]. Smokers who switched to HTP use had improved pulmonary function parameters (forced vital capacity (FVC) and forced expiratory volume in one second (FEV1)), physical exercise capability (assessed using the six-minute walking test), and improved ‘metabolic syndrome parameters’ (waist circumference, HDL-C levels, and systolic blood pressure) than smokers who continued cigarette use at each time-point investigated.

A separate article reported a longitudinal cohort study that aimed to investigate the potential changes in smoking habits after the introduction of HTPs to the Japanese commercial marketplace and their effect on FEV1 decline associated with aging [155]. Study participants, in relation to FEV1 analysis, consisted of a resident population (n=2,612 with a mean age of 67.7 years) with FEV1 measurement conducted from 2012 to 2014 (baseline) and 2018 to 2019 (follow-up). Participants were classified as never-smokers, former smokers, exclusive HTP users, exclusive cigarette smokers, or dual product users (of both HTPs and cigarettes). Differences in annual FEV1 changes between product use groups from baseline to follow-up were examined in the resident population. Using exclusive cigarette smokers as the reference group, exclusive HTP users (+6 ml; 95% confidence intervals of -11 ml to +23 ml) and dual product users (-16 ml; 95% confidence intervals of -34 ml to +2 ml) had non-statistically significant differences in annual FEV1 changes after adjustment of gender, age, height, FEV1 at baseline, and number of cigarettes smoked per day at baseline. Adjustment of results for gender, age, height, FEV1 at baseline, and the number of cigarettes/consumables smoked/used per day at follow-up produced comparable results for exclusive HTP users (+4 ml; 95% confidence intervals of -15 ml to +24 ml) and dual product users (-11 ml; 95% confidence intervals of -33 ml to +11 ml) when compared to exclusive cigarette smokers. The authors provided no statistical analyses using never-smokers, rather than exclusive cigarette smokers, as the reference group.

In the most recent study, as described previously, HTP use had no statistically significant effect on exhaled carbon monoxide levels or pulmonary function parameters (n=7 when baseline levels were compared to those measured 30 minutes after product use [144]. The exhaled nitric oxide fraction (FeNO) increased to a statistically significant extent from 12.8±5.5 ppm before product use to 14.3±6.2 ppm 30 minutes later. The currently available experimental evidence suggests that HTP use is associated with less profound effects on the respiratory system when compared to cigarette use. 

Respiratory System: Effects on Chronic Obstructive Pulmonary Disease (COPD)

Two reviews relating to the effects of HTP use on the incidence and severity of COPD were identified for inclusion in the update [156,157], in addition to a study utilizing a population modeling approach (which will be discussed separately later in this section). The first identified review concluded that “the current research on the impact of electronic cigarettes and HTPs on COPD patients’ health is limited, and more high-quality studies are needed to draw definitive conclusions” and that “while electronic cigarettes and HTPs may offer some benefits in reducing harm from cigarette smoke, their long-term effects on COPD patients’ health are still unclear” [156]. It should be noted, however, that the authors of this review were the authors of the sole study relating to HTPs and COPD detailed in the original article [158], and this was the sole clinical study relating to HTPs that they discussed in detail in their review. The second identified review concluded that “the congruence of recent scientific findings indicates that adult smokers who completely switch to HTPs could have a lower risk of COPD development and progression than those who continue smoking. However, the paucity of studies on this specific topic limits the strength of this conclusion. Further epidemiological studies, additional follow-up of real-world data, and prospective clinical trials are needed to understand the impact of switching to HTPs on health outcomes in COPD” [157]. The currently available evidence is not sufficient to draw any conclusions in regard to the effects of HTP use on COPD.

Immune System

A single clinical study on male participants was identified that investigated the potential effects of HTP use on aspects of the innate immune system. In the study, the secretion rates of salivary lactoferrin and salivary lysozyme (two antimicrobial proteins present in saliva) were determined in exclusive HTP users (n=32), exclusive cigarette smokers (n=17), dual product users (n=14) and non-product users (n=149) [159]. The presence of periodontal diseases was an exclusion criterion for the study. A reduction in secretion rates was suggested as indicating an adverse effect on the salivary glands. The secretion rate was an experimental measure of the amount of saliva secreted by participants onto a piece of cotton wool over a five-minute period. The median saliva secretion rates for exclusive HTP users (1.0 ml/minute) and exclusive cigarette smokers (1.1 ml/minute) were significantly decreased when compared to non-product users (1.4 ml/minute) but not dual product users (1.1 ml/minute). There were no statistically significant differences in the concentrations of salivary lactoferrin or salivary lysozyme between any of the product use groups. The authors noted a “possible adverse effect of a heat-not-burn cigarette on the amount of lactoferrin and lysozyme released into the oral cavity." The clinical significance and relevance of the experimental methodology employed to the real-world product use scenario remain to be determined. The currently available evidence is not sufficient to draw any conclusions in regard to the effects of HTP use on the immune system. A single clinical study on male participants was identified that investigated the potential effects of HTP use on aspects of the innate immune system. In the study, the secretion rates of salivary lactoferrin and salivary lysozyme (two antimicrobial proteins present in saliva) were determined in exclusive HTP users (n=32), exclusive cigarette smokers (n=17), dual product users (n=14) and non-product users (n=149) [159]. The presence of periodontal diseases was an exclusion criterion for the study. A reduction in secretion rates was suggested as indicating an adverse effect on the salivary glands. The secretion rate was an experimental measure of the amount of saliva secreted by participants onto a piece of cotton wool over a five-minute period. The median saliva secretion rates for exclusive HTP users (1.0 ml/minute) and exclusive cigarette smokers (1.1 ml/minute) were significantly decreased when compared to non-product users (1.4 ml/minute) but not dual product users (1.1 ml/minute). There were no statistically significant differences in the concentrations of salivary lactoferrin or salivary lysozyme between any of the product use groups. The authors noted a “possible adverse effect of a heat-not-burn cigarette on the amount of lactoferrin and lysozyme released into the oral cavity." The clinical significance and relevance of the experimental methodology employed in the real-world product use scenario remain to be determined. The currently available evidence is not sufficient to draw any conclusions in regard to the effects of HTP use on the immune system.

Urologic and Reproductive Health

A literature review was identified that aimed to assess the impact of two NGPs, EVPs and HTPs, on urologic health [123]. The authors reviewed articles published up to April 2021 and categorized them based on the potential impact of NGP use on erectile dysfunction, semen quality, lower urinary tract symptoms, and genitourinary malignancies, with a direct comparison made with cigarette use in each instance (information relating to genitourinary malignancies is detailed separately in the biomarker section of this review). With respect to erectile dysfunction, the authors noted that “the current literature lacks data comparing the rates of erectile dysfunction between e-cigarette or heat-not-burn product users and combustion cigarette smokers or non-smokers, making it impossible to draw reliable conclusions in regards to the possible impact of these alternative tobacco products on erectile function." With respect to semen quality, the authors noted that “up to date, no studies regarding the impact of heat-not-burn tobacco products on semen quality have been published in the literature." With respect to lower urinary tract symptoms, the authors noted that “no evidence regarding an association between the use of heat-not-burn products nor electronic cigarettes (and lower urinary tract symptoms) has been published in the literature to date” and that “no definitive data has shown a link between e-cigarette or heat-not-burn product use and lower urinary tract symptoms." It is readily apparent that studies on the impact of HTP use on urologic health are currently lacking, and, as such, no definitive conclusions regarding the effect of HTP use on urologic health can be made at this time.

An observational study was identified that attempted to evaluate the impact of cigarette use and NGP use on the quality of oocytes retrieved from women undergoing intracytoplasmic sperm injection (ICSI) cycles as part of *in vitro* fertilization (IVF) treatments [160]. The prospective observational study included a total of 410 women referred to a specialist fertility clinic over a three-year period from 2019 to 2022. The cohort of women investigated was stratified into non-smokers (n=207; 51.5%) and ‘smokers’ (n=203; 49.5%) based on their responses to a questionnaire. The ‘smokers’ group consisted of cigarette smokers (n=103; 51%), EVP users (n=60; 29%), and HTP users (n=40; 20%). Women who smoked cigarettes or used their NGP more than ten times per day (but were not dual product users) were included in the study. The non-smoker's group included former smokers who had stopped for at least a year prior to involvement in the study. When the ‘smoker’ group was compared to the non-smoking group, the ‘smoker’ group had statistically significant decreases in the number of oocytes retrieved per patient (5.21±0.9 and 6.55±3.5, respectively) and in the overall fertilization rate (68.12±2.21% and 72.16±3.05%, respectively) and a statistically significant increase in the number of empty zona pellucida (EZP) oocytes (non-viable and non-functional oocytes) (0.51±0.1 and 0.2±0.1, respectively). Four other oocyte parameters showed no statistically significant differences between the non-smoking and ‘smoking’ groups. Further statistical analyses between cigarette smokers and HTP users found no statistically significant differences between the two groups for seven oocyte parameters. However, the study did not conduct a statistical analysis for results between EVP users and HTP users or between the non-smoker group and HTP users (with cigarette smokers and EVP users being excluded). The authors concluded that “while still little is known regarding the possible impact of the electronic cigarette and the heat-not-burn (products on the) reproductive system, our results link to a possible negative impact on oocyte parameters in ICSI cycles.”

Pregnancy: Epidemiological Studies

No epidemiological studies involving pregnant women were identified in the original review [11]. Four epidemiological studies have been subsequently identified for inclusion in the update, which investigated the potential association between HTP use in pregnancy and maternal and/or fetal health endpoints. 

In the first study, the association between HTP use during pregnancy and hypertensive disorders of pregnancy (HDP) and low birth weight (LBW) was investigated using data from a nationwide web-based survey in Japan [161]. A total of 558 post-delivery and 365 currently pregnant women were investigated in October 2020. The prevalence of ever and current HTP use was 11.7% and 2.7% in post-delivery women and 12.6% and 1.1% in currently pregnant women, respectively. Among currently pregnant women who were former cigarette smokers, 4.4% (n=4 of 91) were current HTP users. There were no statistically significant increases in the risk of HDP (odds ratio of 2.78; 95% confidence intervals of 0.84 to 9.15) or LBW (odds ratio of 2.08; 95% confidence intervals of 0.80 to 5.39) for HTP ever users when compared to never HTP users after adjustment for a range of confounding factors. These null associations for ever HTP use, when compared to never HTP use, persisted when analyses were restricted to never cigarette smokers (n=421) and former smokers (n=137). The authors concluded that “in Japan, the incidence of HTP use exceeded 10% among pregnant women, and HTP smoking may be associated with maternal and neonatal risks." However, their second conclusion was reached based on odds ratios adjusted solely for age; further adjustment for additional confounding factors, including cigarette smoking, removed this effect, as shown above.

The second study investigated the association between HTP use during pregnancy and ‘small for gestational age’ (SGA) outcomes in a nationwide cross-sectional study in Japan [162]. The study included 5,647 post-delivery women, all of whom had singleton pregnancies. The post-delivery women were divided into four groups based on self-reported data: lifetime never-smokers (n=4,144), former cigarette smokers prior to pregnancy (n=1,274), and current exclusive users of either HTPs (n=102) or cigarettes (n=127) during pregnancy. Information on the prevalence of SGA, defined as birth weight and height below the 10th percentile, was obtained from the Maternal and Child Health Handbooks of the women. Among the women, the prevalence of exclusive HTP use during pregnancy was 1.8%. The prevalence of SGA among exclusive HTP users during pregnancy and lifetime never-smokers was 5.9% (n=6) and 2.7% (n=111), respectively. The odds ratio for SGA was not significantly increased for exclusive cigarette smokers during pregnancy (odds ratio of 1.95; 95% confidence intervals of 0.81 to 4.67) when compared to lifetime never-smokers, while the odds ratio for exclusive HTP users during pregnancy was (odds ratio of 2.50; 95% confidence intervals of 1.03 to 6.05). The authors concluded that “in Japan, HTP smoking during pregnancy may be associated with an increased risk for SGA." However, it should be noted that this conclusion is based on a very small number of SGA cases among exclusive HTP users (n=6), which may limit the generalisability of the result.

The third study investigated whether or not HTP use during pregnancy was associated with an increased prevalence of allergies in infants [163]. A total of 5,688 pairs of postpartum Japanese women and their infants (aged below three years of age) were investigated in a web-based cross-sectional survey conducted in 2021. A total of 5.5% of women used HTPs three months before pregnancy, 2.4% of the women used HTPs during pregnancy, and 7.8% of their infants were medically diagnosed with at least one of three allergy-related diseases (asthma, rhinitis/conjunctivitis, or atopic dermatitis). Use of HTPs three months before pregnancy was not associated with a statistically significant increase in the prevalence of allergic diseases for either former (prevalence ratio of 1.35; 95% confidence intervals of 0.98 to 1.86) or current (prevalence ratio of 1.41; 95% confidence intervals of 0.97 to 2.05) HTP users who did not use cigarettes during pregnancy or after birth when compared to never users. The use of HTPs after birth was associated with an increased prevalence of allergy-related diseases in former HTP users but not in current users. The authors concluded that “maternal heated tobacco product smoking during pregnancy is associated with allergy in the offspring.”

The fourth study reported a retrospective, single-site study that compared pregnant women who used HTPs (n=138) with those who smoked cigarettes (n=120), used EVPs (n=114), or reported no-product use (n=270) in regard to maternal and neonatal outcomes [164]. The 642 women were enrolled over a one-year period between July 2021 and July 2022. The biochemical analysis, which included eight separate endpoints, did not indicate any significant differences between any of the four groups. Of all product use groups, cigarette smokers demonstrated the most pronounced difference between the two methods of calculating gestational age (either according to the last menstrual period or based on ultrasound). This was the case in both trimesters. The authors concluded that “the comparison of the data obtained between conventional cigarette smokers and HTP users underlies the greater danger of conventional cigarettes." However, it should be noted that the authors did not conduct any statistical analyses between the quantitative results for the four product use groups investigated, and therefore no definitive conclusions can be reached in regard to the investigated endpoints. The currently available evidence is not sufficient to draw any conclusions in regard to the effects of HTP use in pregnancy.

Oral/Dental Health

A number of studies or reviews were identified that detailed the potential effects of HTP use on several oral- or dental-related endpoints. The first study assessed the potential effect of NGP use on halitosis [165]. The article proposed a three-layer approach combining the use of breath analysis techniques and multi-omics analysis to define the interactions between oral bacterial species and their role in halitosis both *in vitro* and *in vivo*. Such an approach was reported as being able to compare the effects of different nicotine-containing products on oral bacteria and quantify their impact on halitosis. With respect to the effect of HTP use on bacterial species present in oral samples collected from HTP users, equivocal results have been reported. While one study reported differences in the composition of the oral microbiome between cigarette smokers and HTP users [166], a separate study found no significant changes [167].

A systematic review aimed to evaluate and possibly differentiate the effects of cigarettes, HTPs, and EVPs on periodontal and peri-implant health status [168]. However, none of the eighteen studies that met its inclusion criteria provided results in relation to HTPs. The study did, however, identify a study protocol that related to a six-month randomized clinical study into the effects of HTP use on periodontitis treatment outcomes [169]. The results of this clinical study, published after the systematic review of D’Ambrosio et al. [168], reported equivocal results [167]. In the clinical trial, cigarette smokers with generalized periodontitis were randomized to continue smoking or were switched to using an HTP (with no treatment group of study participants being randomized to smoking cessation). The participants underwent standard treatment for periodontitis for up to eight weeks, with dental assessments being conducted at baseline and at three and six months after the first treatment. The results of the study indicated that when HTP users and cigarette smokers received the same treatment for periodontitis, there was no statistically significant difference in the treatment outcome for either group of subjects. The authors concluded that “further studies would be required to better understand the long-term potential benefit of THS (tobacco heating system) on oral health.”

A study protocol was identified describing a planned study that aims to investigate the differences in salivary microRNA (miRNA) expression profiles between HTP users, cigarette smokers, and non-product users [170]. The miRNAs have been proposed as potential biomarkers useful for the early diagnosis of oral diseases. A separate review on the effects of cigarette, EVP, and HTP exposure on miRNA-mediated gene expression concluded that “differential expression of miRNAs was reduced in aerosol from e-cigarettes and tobacco heating products when compared to cigarette smoke. However, there was a significant alteration of some miRNA expression when compared to air controls in both electronic cigarettes and tobacco heating products” [171].

Six studies identified in our original review [11] investigated the effects of HTP use on the discoloration, staining, and color stability of a range of dental hard tissues and dental materials, including dental resin composites, enamel blocks, and artificial dentures. As concluded in the original review, in each study identified, the HTP under investigation was found to result in significantly less discoloration to the dental resin composites, bovine enamel preparations, or artificial dentures than mainstream smoke from cigarettes. Two systematic reviews [172,173] and a single experimental study [174] were identified for inclusion in this update, which detailed the effects of HTP use on staining/color stability of a range of dental hard tissues and dental materials. The first systematic review identified a total of 27 articles that met its inclusion criteria and concluded that “tobacco smoke causes dental staining. There was limited evidence that e-cigarettes and HTPs did cause dental staining that was less intense than that caused by traditional tobacco products” [172]. The second systematic review, relating specifically to dental resin composites, identified a total of 13 articles that met its inclusion criteria and concluded that “resin-based composites are subjected to irreversible color change if exposed to smoke. Electronic cigarettes (both electronic nicotine delivery systems and tobacco heating systems) induce less color change that can be recovered with repolishing or whitening products” [173]. The experimental study produced comparable findings indicating that HTP aerosols reduced tooth discoloration potential compared to mainstream cigarette smoke [174].

The currently available evidence is not sufficient to draw any conclusions in regard to the effects of HTP use on oral/dental health. However, experimental data suggests that exposure to HTP aerosol produces less pronounced tooth staining than exposure to mainstream cigarette smoke under laboratory conditions.

Skin Surface Staining: Clinical Studies

A single clinical study was identified, which investigated the potential effects of HTP use on skin staining [175]. Cigarette smoking was reported by the authors to result in localized staining and aging of the skin in smokers. The effects of HTP use on users’ skin remain unknown, and a study was undertaken to investigate this.

The study aimed to determine whether using an EVP or an HTP resulted in skin staining or activation of biomarkers associated with oxidative stress. Eight areas were identified on the backs of ten study participants. Two of the eight areas were used for air controls, and two areas were exposed to 32 puffs of mainstream cigarette smoke, either EVP aerosol or HTP aerosol, which were delivered to the skin using a 3 cm-diameter exposure chamber. Skin staining was measured using a chromameter, while three biomarkers associated with oxidative stress (squalene (SQ), squalene monohydroperoxide (SQOOH), and malondialdehyde (MDA)) were measured in sebum samples collected from the skin areas. Mainstream cigarette smoke significantly increased skin staining, SQOOH and MDA levels, and the ratio of SQOOH to SQ. Exposure to HTP aerosol produced results comparable to those of both EVP aerosol exposure and untreated air controls, while cigarette smoke exposure significantly increased skin staining. The authors concluded that “the data generated in this pilot clinical assessment suggest that tobacco heating products and electronic cigarettes may have both hygiene and cosmetic benefits for consumers who switch from cigarettes to exclusive use of tobacco heating products or electronic cigarettes." The findings of this clinical study using human participants were comparable to those reported previously by the same authors using porcine skin *ex vivo* [176]. The currently available evidence suggests a reduced effect of HTP aerosol on skin staining when compared to exposure to mainstream cigarette smoke. However, further research is required. 

Medical Case Reports Related to Heated Tobacco Products

Three medical case reports relating to HTPs were identified for inclusion in the update. Two described health effects reported to be associated with the use of HTPs, while the third described a case of accidental ingestion of an HTP consumable in an infant.

The first medical case report detailed the presence of a lesion on the patient’s left lower lip, which was accompanied by burning pain and was reported to have developed four to five months after starting daily use of the HTP [177]. The patient had reportedly switched to HTP from cigarette use. The lesion decreased in size and became less painful after appropriate pharmaceutical treatment.

The second medical case report detailed the development of acute eosinophilic pneumonia (AEP) in a 22-year-old Japanese woman [178]. Two weeks before the onset of symptoms, the woman, who was reported not to be a smoker up to this point, had initiated the use of HTPs. No further details were provided in the medical case report other than that she initially started smoking six cigarettes per day and then subsequently increased her consumption to fifteen cigarettes per day just before the onset of symptoms. No information was provided in relation to the commercial brand used. The patient was hospitalized for a total of nine days. She received methylprednisolone steroid treatment, after which her condition improved rapidly. At the six-month follow-up, the patient was not using HTPs; there was no recurrence of AEP and no long-term sequelae.

The third medical case report detailed a case of accidental ingestion of an IQOS heatstick by a ten-month-old infant [179]. The infant vomited in the hospital prior to the planned removal of the consumable by gastric lavage, which was successful in the expulsion of the consumable. The infant was discharged from the hospital twenty-four hours later, without sequalae.

The evidence provided from medical case reports is anecdotal in nature and is inherently biased given the selection of patients and the lack of a suitable control group. In addition, it is possible that the reported health effects may be due to factors not reported in the article or to factors not reported by the patients to medical staff. Finally, it should be noted that the number of medical case reports relating to HTPs is extremely small compared to the number of HTP users.

Population Health Impact Modeling of Heated Tobacco Product Use

One study detailing the population health modeling of HTP use was identified. The use of modeling approaches may be suitable for assessing the effects of NGP use at the population level for extended time periods. A separate observational study examined the effects of HTP use on the incidence and severity of COPD and ischaemic heart disease (IHD) using a population-level assessment of Japanese hospital admission data over a nine-year period [180]. In this study, a time-trend analysis was conducted using data from the Japanese Medical Data Center (JMDC) database. The numbers of patients hospitalized for both COPD and IHD were retrieved from the JMDC database for time periods relating to before (2010 to 2014) and after (2014 to 2019) the introduction of HTPs to the Japanese market (2014). An interrupted time-series analysis was then used to investigate whether or not the introduction of HTPs into the Japanese market was associated with a statistically significant reduction in hospitalizations for COPD and IHD after adjustment for age, gender, season, and flu vaccination rates. A statistically significant reduction in the number of hospitalizations for both COPD and IHD was observed using data from the JMDC database.

The identified modeling study estimated the impact of the hypothetical introduction of two NGPs (HTPs and EVPs) in Germany from 1995 to 2015 on mortality due to lung cancer, COPD, IHD, and stroke in both men and women aged between 30 and 79 years of age [181]. The model employed a null scenario where neither of the NGPs were commercially available and a range of alternative scenarios where they were commercially available. The null scenario predicted 852,000 deaths from cigarette use (equivalent to 42,600 deaths per year over the investigated time frame) and 8.61 million life years lost. In one alternative scenario where everyone had ceased smoking in 1995 without the use of any NGPs, the model estimated that these values for deaths and life-years lost would have decreased by 217,000 and 2.88 million, respectively, compared to the values estimated by the null scenario. In an additional alternative scenario where everyone switched immediately to HTPs from cigarettes, the model estimated that these values for deaths and life-years lost would have decreased by 159,000 and 2.06 million, respectively, compared to the values estimated by the null scenario. The authors concluded that “deaths from cigarette smoking could be substantially reduced not only by cessation but additionally by switching to reduced-risk products.”

The available evidence from modeling data suggests that under theoretical conditions achieved using defined parameters and assumptions, switching to HTP use would lead to significant decreases in hospital admissions for cardiovascular diseases and a decrease in smoking-related deaths. Further additional studies using data from other geographical populations are required to provide a more detailed analysis of modeling outcomes.

Summary of Health Effects Data

The experimental and epidemiological results discussed in this section of the review do not provide consistent evidence to suggest that HTP use is associated with unfavorable cardiovascular and respiratory effects. In comparison, cigarette use consistently demonstrated unfavorable results in those studies where both product types were investigated. At a population level, the introduction of HTPs has been associated with decreased hospital admissions for both COPD and IHD using real-world data obtained from databases independent of the authors.

While data are now available in relation to the effects of HTP use during pregnancy, effects on the immune system, and oral/dental health, which were not available for inclusion in the original review, it is currently insufficient to draw any firm conclusions in regard to whether or not HTP use produces unfavorable outcomes. Finally, it remains the case, as indicated in the original review, that no novel health effects have been associated with HTP use that have not been previously reported with the use of tobacco and/or nicotine-containing products.

Further research is warranted into the health effects associated with HTP use. It should be noted that a significant number of the studies detailed involved Japanese participants, and it would be informative to obtain additional results from other populations. Prospective cohort epidemiological studies and case-control studies involving HTP users and non-users could be conducted to provide long-term epidemiological evidence in relation to the potential health effects.

Heated tobacco products, indoor air quality, and bystander exposure

This section of the review will discuss those articles that have investigated the effects of HTP use on indoor air quality (IAQ) and the potential health effects associated with bystander exposure. These articles cover four main areas: the chemical characterization of indoor environments, biomarker studies using individuals exposed to such environments, the potential cancer risk associated with exposure to such indoor environments, and the potential health effects associated with exposure to such indoor environments.

Chemical Characterization Studies

Four studies relating to the potential effects of HTP use on indoor air concentrations of chemical constituents were identified in this update [182-185]. Two of the studies did not provide a direct comparison with cigarette use in their experimental designs, so they will not be discussed further [182,183]. An additional study was identified that investigated the potential use of moss as a bioindicator of IAQ and reported on levels of metals present in moss after HTP, EVP, and cigarette use in a domestic environment [186]. However, the lack of an appropriate control in the experimental methodology of this study precludes further discussion of the reported results.

In the first study, the effect of the use of an HTP, commercialized as Ploom X, and two hybrid devices was investigated in an environmentally controlled chamber [184]. Two separate experimental conditions, which were simulations of a restaurant and a domestic residence, were investigated, with cigarette use and no product use (with the presence of study participants in the chamber) acting as positive and negative controls, respectively. The indoor air concentrations of 48 chemical constituents representing a range of tobacco-specific and general IAQ-related markers were quantified. Under simulated restaurant conditions, 25 of the 48 chemical constituents were quantified at levels above either the limit of detection or the limit of quantification of the analytical methods used with HTP. Eight of the 48 chemical constituents showed statistically significant increases in their indoor air concentrations when compared to the negative control (participants present in the chamber with no product use). Only one of these chemical constituents, glycerol, showed a higher indoor air concentration with HTP use when compared to the positive control (participants present in the chamber using cigarettes); the remaining seven showed lower indoor air concentrations. With respect to simulated residential conditions, 24 of the 48 chemical constituents were quantified at levels above either the limit of detection or the limit of quantification of the analytical methods used with HTP. Three of the 48 chemical constituents showed statistically significant increases in their indoor air concentrations when compared to the negative control (participants present in the chamber with no product use). The author noted that glycerol showed a non-statistically significant increase in indoor air concentration with HTP use when compared to cigarette use. The authors concluded that “compared with the presence of people, the concentrations of some constituents were actually increased when using heating tobacco products under both environmental conditions and simulating restaurant and residential spaces. However, the constituent concentrations were lower than those obtained by cigarette smoking, except for propylene glycol and glycerol (with this relating particularly to the hybrid devices), and below the exposure limits for constituents in the air, as defined by air quality guidelines or regulations. Based on these data, the use of heating tobacco systems in appropriate indoor environments has less impact compared to conventional cigarettes.”

In the second study, the effect of HTP use on the indoor air concentrations of several chemical constituents (six volatile organic compounds, nine aldehydes, nanoparticles of size range 16.8 to 552.3 nm, and particulate matter of size range 0.5 to 10 µm) in an office room was determined [185]. Three separate HTPs were investigated (Glo, Lil, and IQOS) in addition to three cigarettes (This Plus, Parliament Aqua 5, and Dunhill). Each experiment was conducted over a two-hour period, with a study participant taking 12 puffs of each product over a five-minute period as soon as they entered the office room. After product use, the study participants left the room, and air sampling was conducted for one hour. Levels of volatile organic compounds, aldehydes, nanoparticles, and particulate matter present after HTP use were significantly lower than levels present after cigarette use. The authors noted that “although the amount generated from heat-not-burn products was small compared to those from conventional cigarettes, various kinds of volatile organic compounds, aldehydes, nanoparticles, and particulate matter were produced, and these were confirmed to affect indoor air quality.”

Biomarker Studies Related to Exposure of Bystanders to Emissions Produced by Heated Tobacco Product Use

Two studies were identified that quantified levels of BOE and BOPH in bystanders exposed to emissions produced by HTP use [187,188]. The first study aimed to correlate the levels of nicotine metabolites present in the urine of women and their children with HTP use by their husbands [187]. A total of 41 families, including a total of 129 participants, were recruited into the study. The study population consisted of nine fathers who exclusively smoked cigarettes, their non-smoking spouses (n=9), and their children (n=18) (Group A), twenty-two fathers who exclusively used HTPs, their non-smoking spouses (n=22), and their children (n=44) (Group B), and ten fathers who were non-users of either product, their non-smoking spouses (n=10) and their children (n=26) (Group C). A single urine sample was collected from each participant. No information was provided in relation to which brands of HTPs were used by the men who self-reported use. The non-smoking spouses and children in Group A had an average total urinary nicotine metabolite concentration of 0.0107 nmol/mg creatinine; the non-smoking spouses and children in Group B had an average total urinary nicotine metabolite concentration of 0.0058 nmol/mg creatinine; and the non-smoking spouses and children in Group C had an average total urinary nicotine metabolite concentration of 0.0012 nmol/mg creatinine. Statistical significance was observed for the differences between all three groups, indicating that average total urinary nicotine metabolite levels quantified in those family members exposed to emissions from HTP use in indoor environments were significantly greater than those not exposed but significantly lower than those exposed to environmental tobacco smoke from cigarettes. The levels of nicotine metabolites quantified in the urine of spouses and children of smokers and HTP users were approximately nine times and five times higher, respectively, than those seen in the urine of spouses and children of non-smokers.

The second study quantified levels of total nicotine equivalents (TNE) and NNAL, as well as 8-OHdG and m7Gua (markers of oxidative stress and DNA damage, respectively), in the urine of non-smoking individuals exposed either to ETS or emissions from HTP use in the home [188]. The study investigated a total of 746 participants who were stratified by their self-reported exposure(s) into those with no exposure (n=562), those exposed to ETS alone (n=64), those exposed to emissions from HTP use (n=46) and those exposed to both (n=4). No information was provided in relation to which brands of HTPs were used in those households where HTP use was present. Quantified TNE values were equivalent in those exposed to either ETS or emissions from HTP use and were significantly elevated compared (2.00 ng/mg creatinine; p<0.01) to those with no self-reported exposure (0.96 ng/mg creatinine). Urinary NNAL levels were significantly elevated in those exposed to either ETS (2.30 pg/mg creatinine) or emissions from HTP use (2.50 pg/mg creatinine) and were significantly elevated compared to those with no self-reported exposure (1.60 pg/mg creatinine). Urinary m7Gua levels were equivalent in those exposed to either ETS or emissions from HTP use and were significantly elevated compared (10.0 µg/mg creatinine; p<0.05) to those with no self-reported exposure (8.40 µg/mg creatinine). No statistically significant differences were observed for 8-OHdG levels between any of the groups investigated.

Assessment of Cancer Risk Associated With Bystander Exposure

A single study was identified that attempted to quantify the cancer risk associated with bystander exposure to emissions from HTPs and compare this to the cancer risk associated with ETS exposure [189]. The described methodology used polyurethane foam facemasks worn by non-smoking volunteers present in the vicinity of smokers to collect emissions released by their smoking activity. Chemical analyses were then conducted on the facemasks to determine the concentration of 54 ETS-related compounds with these quantitative results used in an analysis of estimated cancer risk. Experimental studies were conducted with cigarettes, an HTP (IQOS), and an EVP. The authors reported that the total cancer risk indices associated with NNN, NNK, 4-ABP, and 2-NA present in ETS from cigarettes were significantly higher than those quantified with either the EVP or HTP. However, the concentrations of the ETS-related compounds present on the facemasks worn by volunteers and the deposition of ETS-related compounds do not necessarily reflect the level of absorption into the human body.

Potential Health Effects in Bystanders After Exposure to Emissions Produced From Heated Tobacco Product Use

A single epidemiological study, based on a cross-sectional design, was identified that attempted to investigate the association between exposure to emissions produced from HTP use and respiratory symptoms in current non-smokers [190]. The original review [11] identified two surveys that detailed the self-reported frequencies of a range of symptoms alleged to be associated with this exposure scenario. However, neither of these two articles provided epidemiological analyses of their reported results.

The study assessed data from non-smoking Japanese adolescents/adults aged between 15 and 80 years of age. Data on exposure to HTP emissions was self-reported by study participants. Asthma/asthma-like symptoms were regarded by the authors as the primary outcome and persistent cough as the secondary outcome. Of the overall cohort, 31.9% were former cigarette smokers, 4.4% were former HTP users, and 9.6% were former users of other tobacco products. Around 23.4% of the overall cohort self-reported exposure to HTP emissions in the previous year, while 80.6% self-reported exposure to environmental tobacco smoke from cigarettes over the previous month. A total of 9.8% and 16.7% of those who self-reported exposure to HTP emissions reported asthma attacks/asthma-like symptoms, and persistent coughs, respectively. For those who did not self-report exposure, the equivalent prevalence values were 4.5% and 9.6%, respectively. Exposure to HTP emissions was associated with respiratory symptoms after adjustment for confounding factors: a prevalence ratio of 1.49 (95% confidence intervals of 1.21 to 1.85) was determined for asthma attacks/asthma-like symptoms, and a prevalence ratio of 1.44 (95% confidence intervals of 1.21 to 1.72) was determined for persistent cough. The authors concluded that “secondhand aerosol exposure from HTPs was associated with both asthma attacks/asthma-like symptoms and persistent cough." However, the cross-sectional nature of the survey precludes an assessment of causality, so it is not possible to confirm that exposure to ETS occurred before the development of the respiratory symptoms. In addition, data relating to exposure to HTP emissions and respiratory symptoms were based entirely on self-reported responses provided by the survey participants and are therefore subject to misclassification and/or recall bias.

Summary of IAQ Data

The available scientific evidence indicates that a range of IAQ chemical constituent markers are either undetectable or present at markedly lower concentrations when compared to those levels observed with cigarette use within indoor environments under experimental conditions; these are typically comparable to background levels and/or below regulatory IAQ exposure standards. In addition, the levels of BOE and BOPH quantified in bystanders exposed to HTP emissions are significantly lower than those quantified in bystanders exposed to ETS from cigarettes. These conclusions are consistent with those reported in the original review and, therefore, remain valid.

A single study was identified, which reported that exposure to HTP emissions in bystanders was associated with an increased prevalence of self-reported respiratory symptoms [190]. Additional research conducted using longitudinal epidemiological designs, such as case-control or cohort studies, is required to support this observation, as cross-sectional epidemiological studies cannot be used to infer causality between exposure and outcome as both are measured at the same time point.

Overall, the available scientific evidence indicates that bystanders are likely to be at a reduced risk of harm from exposure to HTP emissions when compared to the risk associated with ETS exposure. This observation supports the concept of HTP use as a valid approach to tobacco harm reduction.

Summary and suggestions for future research

With respect to novel data identified for inclusion in this review, several key studies were identified:

The *in vitro* toxicological effects for HTPs were almost without exception reported to be significantly less than those observed either with commercially available cigarettes or with scientific reference cigarettes. This conclusion is different from that reached in the original review, where a significant disparity in regard to the reported findings was observed between those studies published by HTP manufacturers and those studies published by researchers independent of HTP manufacturers. With respect to those articles identified in this update, there was a significant increase in the number of articles published by the latter. Nevertheless, almost all of the studies identified, irrespective of the source of their funding, concluded that HTPs elicited significantly reduced *in vitro* toxicological effects when compared to cigarettes. As noted in the original review, HTPs continue to demonstrate decreased (or absent) mutagenic, genotoxic, and cytotoxic effects when compared to cigarettes in those assays recommended by CORESTA for the *in vitro* toxicological testing of cigarette smoke.

All of the identified studies reporting novel *in vivo* data were conducted by researchers independent of HTP manufacturers. A single study was identified that reported an additional analysis of data from a previously published inhalation study that was conducted by an HTP manufacturer. This observation is in opposition to that noted in the original review, where the significant majority of identified *in vivo* studies had been undertaken and published by manufacturers. It is important to note, however, that the relevance of *in vivo* studies for assessing the effects of HTP aerosols on human exposure is unclear. The strongest evidence for the assessment of HTPs is likely to be derived from actual health outcomes in cohorts of HTP users compared to cohorts of smokers and non-smokers.

Data from the on-going British American Tobacco (BAT) ambulatory clinical study relating to the 360-day time-point was identified, which demonstrated that several biomarkers of potential harm moved in a favorable direction towards those seen in never-smokers for study participants randomized to both HTP use and product abstinence, while levels moved in non-favorable directions for those study participants who continued to smoke cigarettes. The original review noted that insufficient data was available to draw any firm conclusions in relation to biomarkers of potential harm given the short-term data that was then available (90- and 180-day time-points).

An additional medical case report was identified in this update, which suggested that the use of HTPs may be associated with the development of the rare disorder, acute eosinophilic pneumonia. This case report is in addition to the three case reports identified in the original review. However, as noted in the original review, the number of reported cases of acute eosinophilic pneumonia is extremely small compared to the number of HTP users. Furthermore, there remains no data to indicate whether or not the incidence of acute eosinophilic pneumonia secondary to the use of HTPs is higher than that associated with cigarette use.

Novel epidemiological data in relation to the effects of HTP use during pregnancy was identified. However, it is currently insufficient to draw any firm conclusions in regard to whether or not HTP use produces unfavorable outcomes with regard to maternal or fetal or neonatal outcomes, given the variability in results reported by the identified studies. Further research is warranted.

## Conclusions

In keeping with the conclusions of the original review, the significant majority of newly identified articles that provide quantitative data have concluded that HTPs show a favorable tobacco harm reduction potential when compared to cigarettes. Although not entirely risk-free, this reduced risk profile has been indicated based on a substantial decrease in levels of toxicants present in aerosols, lower levels of non-nicotine-related biomarkers of exposure and/or levels of biomarkers of potential harm, lower biological effects in in vitro or in vivo experimental studies, and lower levels of indoor air quality markers in indoor environments.

In addition, it remains the case, as indicated in the original review, that no health effects have been associated with HTP use that have not been previously reported with the use of tobacco and/or nicotine-containing products. As a final observation, while the available scientific evidence suggests a significant reduction in smoking-related risk for cigarette smokers who transition to HTPs completely, stopping the use of all tobacco (and nicotine) products entirely will lead to the greatest overall reduction in risk.
